# Lizards as Model Organisms of Sex Chromosome Evolution: What We Really Know from a Systematic Distribution of Available Data?

**DOI:** 10.3390/genes12091341

**Published:** 2021-08-28

**Authors:** Marcello Mezzasalma, Fabio M. Guarino, Gaetano Odierna

**Affiliations:** 1 Department of Biology, University of Naples Federico II, I-80126 Naples, Italy; fabio.guarino@unina.it (F.M.G.); gaetanodierna@gmail.com (G.O.); 2 CIBIO-InBIO, Centro de Investigação em Biodiversidade e Recursos Genéticos, InBIO, Universidade do Porto, Rua Padre Armando Quintas 7, 4485-661 Vairaõ, Portugal

**Keywords:** evolution, genome, karyotype, sex chromosomes, sex determination, Sauria, Squamata

## Abstract

Lizards represent unique model organisms in the study of sex determination and sex chromosome evolution. Among tetrapods, they are characterized by an unparalleled diversity of sex determination systems, including temperature-dependent sex determination (TSD) and genetic sex determination (GSD) under either male or female heterogamety. Sex chromosome systems are also extremely variable in lizards. They include simple (XY and ZW) and multiple (X_1_X_2_Y and Z_1_Z_2_W) sex chromosome systems and encompass all the different hypothesized stages of diversification of heterogametic chromosomes, from homomorphic to heteromorphic and completely heterochromatic sex chromosomes. The co-occurrence of TSD, GSD and different sex chromosome systems also characterizes different lizard taxa, which represent ideal models to study the emergence and the evolutionary drivers of sex reversal and sex chromosome turnover. In this review, we present a synthesis of general genome and karyotype features of non-snakes squamates and discuss the main theories and evidences on the evolution and diversification of their different sex determination and sex chromosome systems. We here provide a systematic assessment of the available data on lizard sex chromosome systems and an overview of the main cytogenetic and molecular methods used for their identification, using a qualitative and quantitative approach.

## 1. Introduction

Sex determination systems are important drivers of biological diversity and a better understanding of the mechanisms and pathways driving their evolution and the diversification of sex chromosome systems represents a fundamental objective of evolutionary biology.

Two main types of sex determination mechanisms (SDMs) occur in tetrapods: environmental sex determination (ESD), normally in the form of temperature-dependent sex determination (TSD), and genetic sex determination (GSD), which may be under female or male heterogamety [1].

Unlike other amniotes such as crocodiles, mammals and birds, which show evolutionary stable mechanisms of sex determination (in the form of TSD or GSD with male and female heterogamety), turtles and squamates are generally characterized by a remarkable evolutionary plasticity of SDMs (e.g., References [1,2,3,4]). Lizards represent unique model organisms in the study of sex determination and sex chromosome evolution as their high phylogenetic and species diversity is matched by an unparalleled variability of different SDMs and sex chromosome systems. A good number of lizard families are characterized by the co-occurrence of TSD and GSD with female and/or male heterogamety (e.g., References [2,5]). Shifts between different systems can also occur in one generation or between different populations in some species [6,7], identifying lizards as promising model organisms also to better understand the mechanisms and the evolutionary drivers of sex reversal and sex chromosome turnover.

Sex chromosomes are also extremely variable in lizards. Various families include simple and multiple sex chromosome systems, which can emerge from non-homologous macro- and microchromosome pairs in different taxa, covering all the different hypothesized steps of diversification of heterogametic sex chromosome pairs, from homomorphic to heteromorphic and completely heterochromatic chromosomes (e.g., References [8,9,10,11]).

The growing interest in lizard sex determination and sex chromosome systems produced in recent years a sensible increase of the available data on different taxonomic groups (e.g., References [12,13,14,15,16,17]), which might be useful to provide novel insights on the phylogenetic distribution of different systems.

In this review, we provide a synthesis on the main characteristics of the lizard genome and karyotype and discuss the main hypotheses and evidence on the evolution and diversification of their different sex determination and sex chromosome systems. Furthermore, we present a systematic qualitative and quantitative assessment of the available data on sex chromosome systems of non-snake squamates, updating the current knowledge of their phylogenetic distribution, using the evolutionary relationships reported by different studies [18,19,20,21].

Finally, we provide an overview of the main molecular and cytogenetic methods commonly used in the characterization of sex chromosomes in lizards, highlighting their main strengths and limits in the identification of GSD and sex chromosome systems.

## 2. General Features of the Lizard Genome and Karyotype

The lizard genome and karyotype show a remarkable diversity, but some general characteristics are shared between different evolutionary lineages [22,23].

Genome size varies in vertebrates and represents a general indicative feature to assess large scale evolutionary trends in genome evolution. Estimates from flow cytometry (FCM) show that the average genome size of lizards is between 1.03 (in *Chalcides mionecton*, Scincidae) and 3.8 Gbp (in *Anguis fragilis*, Anguidae), a lower value compared to urodeles (9.8–117 Gbp), frogs (2–9.8 Gbp), mammals (average of 3.5 Gbp) and non-squamate reptiles (average of 3.2 Gbp in turtles and crocodiles and 5.0 Gbp in *Sphenodon*), but similar to that reported for birds (average of 1.4 Gbp) [24,25,26].

Different studies also highlighted that genome size in reptiles experienced gradual changes during evolutionary time and that dimensional variations occurred at higher rates in larger genomes [27,28].

The quantity of protein-coding nuclear genes remains almost constant in vertebrates and differences in the genome size are mostly related to different amounts of repetitive DNA [29,30]. Compared to other tetrapods, the lizard genome results also relatively scarce in constitutive heterochromatin and overall shows a lower amount of repetitive DNA [22,31]. The majority of DNA repeats in the lizard genome is constituted by transposable elements (TEs), ranging from 20% to about 60% in different taxonomic groups [31,32,33]. The other most abundant DNA repeats in the lizard genome are represented by satellites and microsatellites, with different families and subfamilies characterized by different length, nucleotide structure and chromosome localization [33,34,35,36,37]. Both TEs and satellite DNA, due to their amplification and transposition activities, are known to be involved in significant modifications of the host genome [38,39,40]. Such changes in the genome structure may promote the onset of inter- and intrachromosome rearrangements [41,42], which in turn may contribute to lineage divergence and speciation [43,44,45,46,47]. These mechanisms likely contributed to generate the currently observable karyological variability in lizards, which display an impressive diversity in chromosome number and morphology [22,48].

Diploidy is the standard karyological condition in lizards, but parthenogenetic triploids are known in Gekkonidae, Gymnophthalmidae, Lacertidae and Teiidae, while tetraploids are extremely rare (reported in Gekkonidae and Teiidae) [2,49,50,51,52].

The diploid chromosome number is highly variable in lizards and it is between 2n = 16 in *Gonatodes tanieae* (Gekkonidae) and 2n = 62 in *Rieppeleon brevicaudatus* (Chamaeleonidae) and *Notobrachia ablephara* (Gymnophtalmidae) [2,53,54].

Two fundamental types of karyotypes have been described in lizards: “asymmetrical” and “symmetrical” karyotypes [55,56]. Asymmetrical karyotypes are composed of chromosomes of two clearly different dimensional classes (macrochromosomes and dot-shaped microchromosomes), while symmetrical karyotypes are composed of a variable number of elements gradually decreasing in length [55,56]. Asymmetrical karyotypes are overall more common in lizards and usually include a prevalence of biarmed (mostly meta- and submetacentric) chromosomes, while symmetrical karyotypes often show a high proportion of telocentric elements [48,54,57].

Overall, karyotypes composed of both macro- and microchromosomes are more common in Iguania, Platynota and most Scinomorpha, while morphologically more uniform karyotypes are generally predominant in Gekkota and Lacertidae (see, e.g., References [11,48,58,59,60]).

Because of their high species diversity and a relatively low number of species with a known karyotype, chromosomal evolution in lizards is still poorly understood compared to that of mammals and birds [61]. Nevertheless, some general trends have been identified in different taxa. An interesting dynamic emerging from several studies is that symmetrical karyotypes may have originated from asymmetrical karyotypes multiple times during evolutionary time, through a succession of chromosomal rearrangements [22,31,61]. It has also been hypothesized that the karyotype of the amniote common ancestor included both macro- and microchromosomes, a configuration shared by birds and squamates [62,63]. Furthermore, karyotypes with 2n = 34–36 chromosomes (composed of 12–16 macrochromosomes and 20–24 microchromosomes) are shared by different evolutionary lineages in squamates (e.g., Helodermatidae, most Iguania and snakes) [9,11,48,57,64]. Other than a similar chromosome number and morphology, these karyotypes present a highly conserved genetic linkage of different macrochromosome pairs and are supposed to represent ancestral conditions in several groups (e.g., References [9,57,61,65,66]).

The comparison of available data on chromosome number, morphology and location of repeated sequences highlights that the mobility of microchromosomes is associated to a good portion of the karyological variability currently observable in lizards, snakes and birds [31,61,63]. For example, in gekkonids and lacertids independent, progressive accumulations of (non-homologous) fusions of microchromosomes led to a reduction of the chromosome number and the formation of small sized macrochromosomes [67]. A similar process has been documented in Iguania, where multiple fusions between ancestral telocentric elements and/or microchromosomes produced an overall reduction of the chromosome number and the formation of biarmed elements [57,68].

However, different evolutionary processes took place in different evolutionary lineages, and an augmentation of the chromosome number trough the fragmentation of larger elements may lead to the formation of karyotypes with a higher chromosome number. This process probably originated the high chromosome number in *R. brevicaudatus* (Chamaeleonidae) and analogous processes have been documented for example in Dactyloidae, Gekkonidae and Polychrotidae [57,69,70,71]. Furthermore, in several squamate lineages a combination of both fusions and fissions probably contributed to the generation of inter- and intrageneric karyological diversity (e.g., References [8,51,72,73]) and the reconstruction of complex pathways of karyotype diversification requires a comprehensive taxon sampling and a mixture of traditional and molecular cytogenetics.

## 3. Rise and Diversification of GSD and Sex Chromosome Systems

The extreme variability of sex determination and sex chromosome systems in vertebrates leaves predictions on their putative ancestral states still indeterminate [61,74,75]. Several studies suggested that TSD is the more likely ancestral condition in both amniotes and reptiles and that transitions to GSD, as well as subsequent reversals to TSD, took place multiple times in different evolutionary lineages (e.g., References [74,76,77]). The opposite hypothesis of ancestral GSD in amniotes cannot be rejected basing on available evidence, but it would require a much higher number of transitions, resulting evolutionary less parsimonious [77]. Furthermore, GSD in amniotes, and particularly in lizards, includes a number of different systems which followed distinct evolutionary pathways, involving non-homologous chromosome pairs [5,14,78,79].

TSD is the only known mechanism of sex determination in crocodiles and in Sphenodon; it occurs along with GSD in turtles and lizards, while its presence has not been documented in snakes [4]. TSD, and more generally ESD, is thought to be favored when particular environmental conditions are beneficial to one sex, while the transition from EDS to GSD would occur after the insurgence of either a favorable mutation to the heterogametic sex or different sexually antagonistic genes [1,80]. The maintenance and the evolutionary success of TSD is thus generally thought to be linked to stable environmental condition, while GSD is favored when the environment is hypervariable and unpredictable or when its variability is inadequate for producing a balanced sex ratio [81]. Recent studies also suggested that TSD may be preferentially retained in long-lived species, while shorter lifespan would favor the emergence of GSD. This would explain the predominance of TSD and GSD in turtles and lizards, respectively [82].

Some lizard species may even show a mixture of characteristics of TSD and GSD among different populations living in different environments [83,84]. In the case of Carinascincus ocellatus (Scincidae), TSD occurs in populations at low altitudes where temperature variations are limited, while GSD has been reported in populations at high altitudes where environmental variability is higher [6]. TSD and GSD (along with the emergence of differentiated sex chromosomes) can thus be fast evolving systems and the transition between different mechanisms of sex determination can be promoted by different selective pressures.

From a cytogenetic point of view, the evolution of differentiated sex chromosomes begins from homomorphic autosomal ancestors, after the insurgence of a sex-determining allele [85,86]. Studies on neo-formed sex chromosomes show that they are mostly pseudoautosomal, homomorphic and often undetectable with standard karyotyping (e.g., References [87,88]). The progressive diversification of the Y/W element may be promoted by the accumulation and fixation of deleterious mutations and recombination suppression in the region containing the sex-determining allele [89,90,91,92]. This is favoured by the accumulation of intrachromosomal rearrangements, eventually leading to the progressive degeneration (loss of coding DNA) and the evolutionary isolation of the heteromorphic chromosome [80,93,94,95,96]. The extreme point of this evolutionary trajectory is the establishment of achiasmy among the heteromorphic sex chromosomes (XY/ZW) [97].

**Figure 1:**
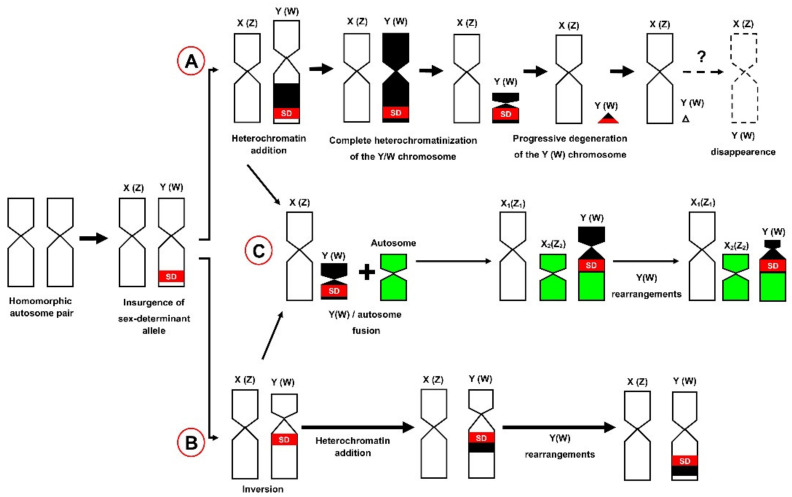
Pathways of sex chromosome diversification in lizards: (**A**) diversification by progressive heterochromatinization, (**B**) diversification by chromosome inversion and (**C**) formation of multiple sex chromosome systems by sex chromosome–autosome fusion.

Two main different pathways are known to possibly lead to a fully differentiated heteromorphic sex chromosome (Figure 1). The first assumes that loss of recombination in the sex determining region is achieved through a progressive addition of heterochromatin (Figure 1A) [91,98]. This can initially produce an increase in size of the heteromorphic chromosome (Y/W) which subsequently goes through a process of progressive degeneration, heterochromatin deletion and dimensional reduction, finally reaching the size of a microchromosome (Figure 1A) [91,98]. However, heteromorphic and/or heterochromatic sex chromosomes in lizards are known to emerge from either macro- or microautosome pairs (e.g., References [9,11,79,99]), and dimensional changes in micro-Y/W may be difficult to detect, especially during early diversification stages.

Repeated sequences are known to play a significant role in sex chromosome evolution and their preferential accumulation on the heteromorphic chromosome may either promote or be promoted by loss of recombination [100,101]. In particular, different satellite, microsatellite and TE families are known to progressively accumulate on heterogametic chromosomes in different squamate lineages, driving processes of heterochromatinization and morphological diversification [60,102,103,104,105,106].

The further step of the progressive diversification of sex chromosomes is the complete loss of the heteromorphic chromosome (Figure 1A). Among tetrapods, this condition has been very rarely observed in mammals [107,108], but has been never documented in reptiles.

A second diversification pathway of the heteromorphic sex chromosome involves the insurgence of an inversion in the homomorphic proto-Y/W (Figure 1B), halting recombination around the sex-determining locus and promoting the progressive degeneration of the heretogametic chromosome (e.g., References [109,110,111]).

Differential DNA methylation has been proposed as a further mechanism possibly contributing to recombination suppression and sex chromosome divergence [112]. This hypothesis involves higher methylation in the region containing the sex-determining allele, inducing chromatin alterations and the accumulation of sex-specific characteristics in the heterogametic sex. However, DNA methylation patterns can be eliminated during development and the role of epigenetic mechanisms on sex chromosome diversification remain controversial [112].

In a significant number of lizard species (see below), the main described pathways of sex chromosome diversification (Figure 1A,B) can also be modified by further chromosome rearrangements leading to the formation of multiple sex chromosome systems (X_1_X_1_X_2_X_2_/X_1_X2_Y_ and Z_1_Z_1_Z_2_Z_2_/Z_1_Z_2_W) (Figure 1C). Such configurations are originated from a Y/W-autosome fusion, producing an odd diploid chromosome complement in the heterogametic sex (e.g., References [10,75,113,114]).

In general, the different steps and pathways of sex chromosome evolution here described (Figure 1) do not follow a common timeframe. Heterogametic chromosomes may quickly diversify and degenerate in some lineages, while they persist over a long evolutionary period of time in other groups as poorly differentiated elements [8,16,71,76,88].

A striking example of rapid sex chromosome diversification has been documented in Dactyloidae, where Anolis and Ctenonotus are characterized by the presence of both macro- and micro-sex chromosomes with a shared origin and an independent insurgence of multiple sex chromosome systems [71,115]. In these clades, repeated fusions between the Y chromosome and non-homologous autosomes have been identified in different closely related lineages, further promoting their high karyological variability [71]. In some cases (e.g., Norops, Dactyloidae, Sceloporus and Phrynosomatidae), fusions between autosomes and sex chromosomes, rather than producing multiple sex chromosome systems may generate single, albeit enlarged pairs, containing distinct pseudoautosomal regions [116,117,118]. In rare cases, sex chromosome diversification can be observed also at intraspecific level in lizards, as in the case of Carinascincus ocellatus, where ecologically distinct populations exhibit different heterochromatinization levels of the Y chromosome [88]. However, poorly differentiated but highly conserved XY chromosomes generally characterize the family Scincidae. The homology of the homomorphic XY pairs in Scincidae resulted to be conserved for at least 85 my, a value comparable to those estimated for the highly differentiated ZZ/ZW sex chromosomes of birds and snakes, highlighting the long evolutionary stability of these elements in various skink genera [16].

## 4. Sex Reversal and Sex Chromosome Turnover

The evolution of heterogametic sex chromosomes has been traditionally considered an irreversible condition in tetrapods [119,120]. This view has been supported by the evolutionary stability of differentiated sex chromosomes in different lineages, leading to the hypothesis that they might represent an evolutionary trap, acting against transitions to new sex-determining systems [121,122,123]. However, squamate reptiles show a high potential for swift transitions between different conditions and in particular cases GSD can be redirected by environmental factors in lizards. The resulting sex-reversed individuals display a sexual phenotype which is the opposite of their genetic sex [124].

Sex reversal has been confirmed in two lizard families, Agamidae and Scincidae, in species showing different sex chromosome systems with either female or male heterogamety. In the Australian agamid *Pogona vitticeps*, sex is normally determined by a sex chromosome system with female heterogamety (ZZ/ZW), showing no homology with other reptiles [5]. However, incubation temperatures > 32° C produce a progeny composed of all females, with about 50% of them presenting the ZZ male genotype [83]. The sex-reversed ZZ females are fertile and mating with normal ZZ males lead to a temperature-dependent sex ratio and the loss of the W chromosome in the following generation [7]. Sex reversal in *P. vitticeps* is controlled by temperature-induced differential expression and alternative splicing of genes of the Jumonji family [125]. Similar mechanisms control TSD in alligators and turtles, suggesting that sex reversal in *p. vitticeps* represents a return to an underlying plesiomorphic condition [125]. In the case of the skink *Acritoscincus duperreyi*, sex is normally determined by a sex chromosome system with male heterogamety (XX/XY), but low incubation temperatures produce a biased sex ratio with XX males [104,126,127]. A similar mechanism has been reported also in another skink, *Eulamprus heatwolei*, which shows a XY system and thermally induced sex reversal at high incubation temperatures [128]. These evidence highlight that transitions from GSD to TSD can occur in one generation in some species and different sex determination systems should be probably threated more as evolutionary variable states than fixed conditions [129].

Considering the lack of experimental testing on temperature-induced sex reversal, analogous mechanisms may be more widespread in lizards than the currently available data are able to suggest. Other cases of potential temperature-induced sex reversal in lizards have been hypothesized in Gekkonidae and Lacertidae, but their effective occurrence in these families needs to be confirmed by further analyses [130].

The evolutionary variability of sex determination systems in lizards goes beyond transitions between TSD and GSD and may involve changes of the sex chromosome pair. Sex-determining loci may shift among different chromosomes or a new master sex-determining gene may emerge in a new pair and an extensive sex chromosome turnover has been documented in Gekkonidae and Pleurodonta [15,131,132]. For example, the family Gekkonidae is supposed to have an ancestral TSD which is retained in some lineages and shows multiple switches to both male and female heterogamety [76,78]. In the genera *Cyrtodactylus*, *Hemidactylus* and *Gekko* male and female heterogamety may also co-occur, suggesting that sex chromosome turnover may also contribute to interspecific diversification [15,76,110,133].

To explain why some sex chromosome systems are maintained over a long evolutionary time period, while others undergo an extensive turnover, a hypothesized model suggested that sex chromosomes regularly go through turnover cycles unless the heteromorphic chromosomes become highly differentiated [76,134]. Once a certain degree of diversification has been achieved the heteromorphic chromosomes can be considered evolutionary stable and sex chromosome turnover becomes extremely unlikely [120].

Sex reversal is also considered a possible driver of non-homologous turnover [130] and more focused analyses on these two co-occurring mechanisms in lizards may provide new insights into complex evolutionary pathways of sex determination in vertebrates.

## 5. Non-homologous GSD and the Amniote Super Sex Chromosome

Sex chromosomes of most vertebrate lineages are non-homologous and emerged independently from different pairs. For example, the conserved ZW pair in birds shows no homology with the XY of mammals or the snake ZW pair [135,136]. Non-homology of sex chromosomes has been demonstrated also between most main lizard evolutionary lineages, as well as within families showing sex chromosome turnover and multiple shifts between GSD and TSD (e.g., References [14,15,78,132]). Nevertheless, recent studies highlighted the occurrence of partial homologies between the Z chromosome of *Gekko hokouensis*, the avian Z chromosome, the X chromosome of giant musk turtles of the genus *Staurotypus*. [74,137,138,139]. Similar homologies have been found between the Z chromosome of *Takydromus sexlineatus* (Lacertidae) and the X chromosome of therian mammals [62,74]. Interestingly, the Z chromosome of Lacertidae is not homologous with sex chromosomes of other reptiles but shares common regions with the X chromosome of different mammal lineages [140].

The super sex chromosome hypothesis suggests that these different chromosomal regions, sharing partial homology across phylogeny, would have been part of an ancient sex chromosome of the amniote common ancestor, which went through fragmentation by means of multiple fissions, originating several different sex chromosome systems in different evolutionary lineages [74,137,138]. Alternatively, it is possible that these homologous regions have been co-opted multiple times independently as sex chromosomes due to their predisposition for a sex-determining function [74,75,132,133,137,138].

## 6. Sex Chromosome Diversity in Lizards: Qualitative and Quantitative Distribution of Available Data

To perform a comprehensive assessment of the available information we checked the data reported in References [48,141], previously published reviews on similar topics [2,3,142,143] and directly searched for publications on different databases (PubMed, Google Scholar, Scopus, ResearchGate and the Reptile Database), using the following keywords in different combinations: sex chromosomes, lizard, squamates, reptiles, sex determination system, genetic sex determination, temperature-dependent sex determination, XY, ZW, TSD and GSD. A second screening was performed checking the data cited in the publications retrieved during the initial search.

**Figure 2:**
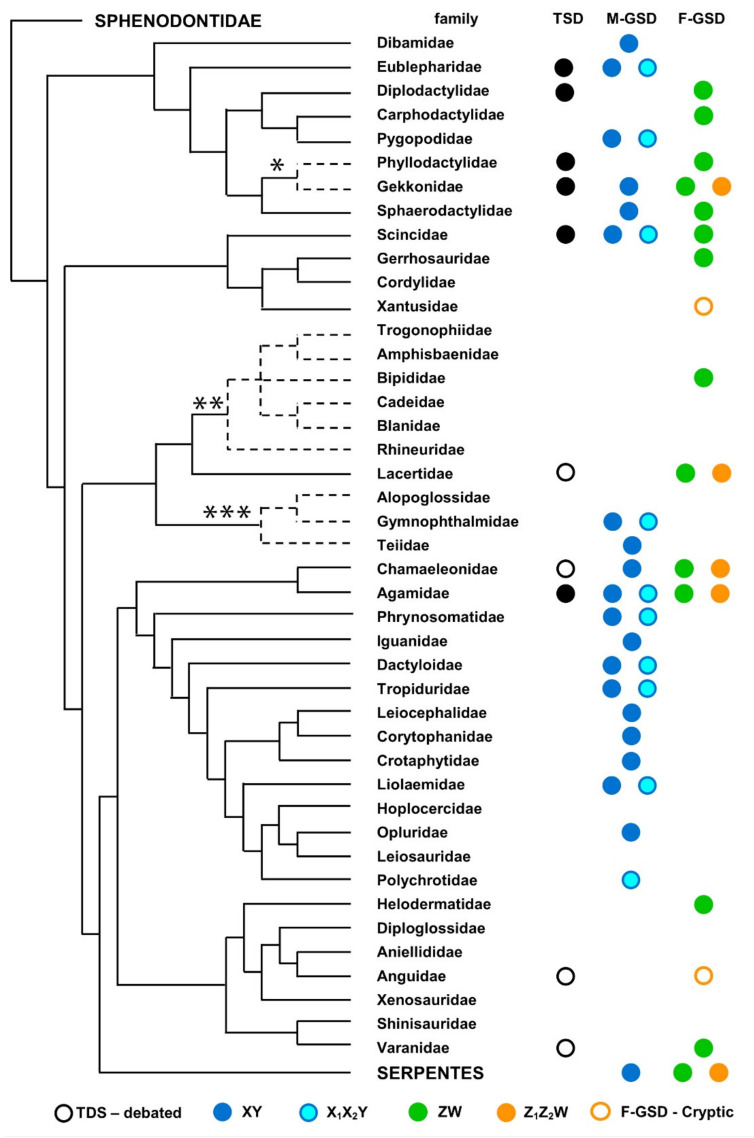
Distribution of TSD and male (M-GSD) and female (F-GSD) genetic sex determination on lizard phylogenetic relationships redrawn from Reference [21]. Dashed lines represent phylogenetic relationships by * Reference [18], ** Reference [19] and *** Reference [20].

Concerning the total number of available data, we counted 376 species with a known sex chromosome system (Supplementary Materials Table S1), more than doubling the number reported in Reference [2] (n = 181). Information on sex determination and sex chromosome systems is currently available on 30 families, while, to date, no data have been reported on 13 families (Figure 2 and Supplementary Materials Table S1).

Overall, known sex determination mechanisms in lizards include TSD and male and female heterogamety with simple (XY/ZW) and multiple (X_1_X_2_Y/Z_1_Z_2_W) sex chromosome systems, showing a complex phylogenetic distribution (Figure 2 and Supplementary Materials Table S1). For completeness, we also listed in the available data a putative X_1_X_2_Y/0W system in *Lygodactylus picturatus*, but the supernumerary chromosome found in this species probably represents a B chromosome, not a sex chromosome system [144].

The available data show that TSD has been documented in 10 lizard families (Figure 2 and Supplementary Materials Table S1), but its occurrence in Chamaeleonidae, Anguidae and Varanidae (which refer to *Chamaeleo chamaeleon*, *Podarcis pityusensis*, *Elgaria multicarinata* and *Varanus salvator*) is considered doubtful (see Reference [128]). In particular, reports of TSD in *C. chamaeleon* is anecdotical [145], and recent studies on this species and on the congeneric *C. calyptratus* evidenced the presence of homomorphic XY sex chromosomes [13,99]. Concerning *p. pityusensis* and *V. salvator*, qPCR studies by Rovatsos et al. [113,146,147,148] evidenced a ZW sex chromosome system, while TDS in *Elgaria multicarinata* was not supported by incubation experiments by Reference [147]. Furthermore, the probable lack of TSD in Lacertidae has been discussed by Rovatsos et al. [149], who found evidence of a conserved ZW system in the family.

When present, TSD is never the only sex-determining systems in any lizard family and always co-occurs with male (Eublepharidae), female heterogamety (Diplodactylidae, Phyllodactylidae and Lacertidae) or both (Gekkonidae, Scincidae and Agamidae) (Figure 2 and Supplementary Materials Table S1).

Either male and female heterogamety have been documented in a high number of different taxa, including a high variability of homomorphic, heteromorphic and multiple sex chromosome systems (Figure 2 and Supplementary Materials Table S1). Male heterogamety is overall more widespread across lizard phylogeny, occurring in 21 families, while female heterogamety has been reported in 13 families (Figure 2 and Supplementary Materials Table S1). Male and female heterogamety are known to co-occur in five families (Gekkonidae, Sphaerodactylidae, Scincidae, Chamaeleonidae and Agamidae) (Figure 2, Table S1), generally involving distinct chromosome pairs and highlighting the occurrence of independent cases of non-homologous sex chromosome turnover and shifts with TSD.

Male heterogamety is the only currently known sex determination system in Dibamidae, Pygopodidae, Gymnophthalmidae, Teiidae and most Iguania families, with the exception of Chamaeleonidae and Iguanidae where it co-occurs with female heterogamety and TSD (Figure 2 and Supplementary Materials Table S1). In particular, male heterogamety in chameleons has been documented to date only in *Chamaeleo chamaeleon* and *C. calyptratus*, using a combination of molecular and cytogenetic techniques which were able to identify a homomorphic XX/XY system [13,99,150]. In turn, female heterogamety is the only known sex determination system in Xantusidae, Bipididae and Helodermatidae (Figure 2 and Supplementary Materials Table S1). Considering the great variability observed among and within different lizard families (Figure 2 and Supplementary Materials Table S1) it is difficult to highlight a clear pathway of phylogenetic diversification of sex determination systems. Nevertheless, male heterogamety is largely predominant in Gymnophthalmoidea and Iguania families, while female heterogamety occurs in more families of Gekkota, Scincomorpha and Anguimorpha (Figure 2 and Supplementary Materials Table S1).

**Figure 3:**
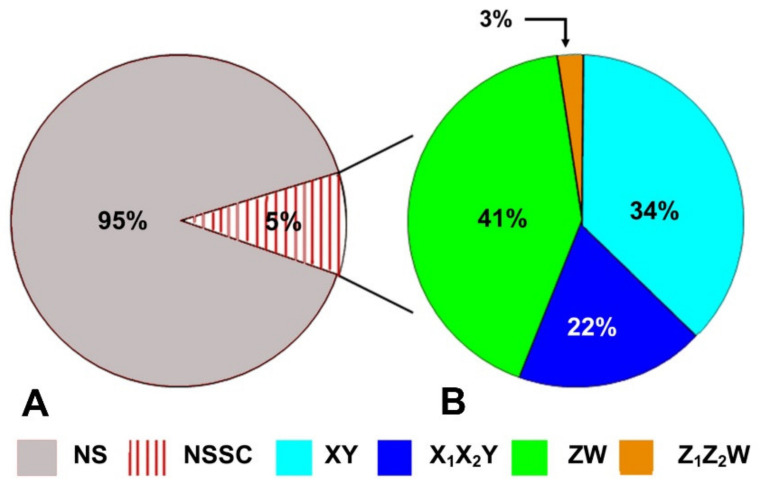
(**A**) NS = Number of described lizard species, NSSC = Number of lizard species with documented TSD or sex chromosome system; (**B**) Relative abundance of different sex chromosome systems in lizards.

It should also be noted that, even if recent studies greatly improved our knowledge on the phylogenetic distribution of sex determination systems among and within different families, the amount of total available data is still very limited compared to their species diversity. Sex chromosome systems are known in just 5% of the total described lizard species (Figure 3A) and, considering just the families with more than ten described species, only Phrynosomatidae, Lacertidae and Varanidae include 20% of described species with a known sex chromosome system.

Quantitatively, ZW is the most common sex chromosome system in lizard, as reported in about 40% of the species so far analyzed (Figure 3B and Supplementary Materials Table S1). However, when considering together simple and multiple sex chromosome system, male heterogamety (XY + X_1_X_2_Y = 56% of the studied species) is overall more common than female heterogamety (ZW + Z_1_Z_2_W = 44%). This is explained by the significant number of multiple sex chromosome systems with male heterogamety (X_1_X_2_Y = 22%), which is considerably higher than the number of multiple sex chromosome systems with female heterogamety (Z_1_Z_2_W = 3%). The current data are unable to provide a conclusive explanation for this strongly unbalanced occurrence of multiple sex chromosome systems, but it has been previously hypothesized that multiple sex chromosomes under female heterogamety would more likely produce an unbalanced sex ratio due to female meiotic drive and should be therefore penalized by selection [151].

Similar ratios of XY/ZW-autosome fusions have been previously reported in squamates reptiles (33% of XY species showed fusions against only 3% of ZW species) and fishes (41% of XY species showed fused sex chromosomes against 5% of ZW species) [152]. A possible explanation proposed by Pennell et al. [152] for the preferential occurrence of Y-autosome fusions is that fusions are slightly deleterious, and that the mutation rate is male-biased or the reproductive sex ratio is female-biased. Even if other evolutionary mechanisms may play a significant role in particular species (e.g., neutral selection, meiotic drive and direct fitness effects), they were not regarded, alone, able to explain the preponderance of Y-autosome fusions observed in distinct vertebrate lineages [152].

**Figure 4:**
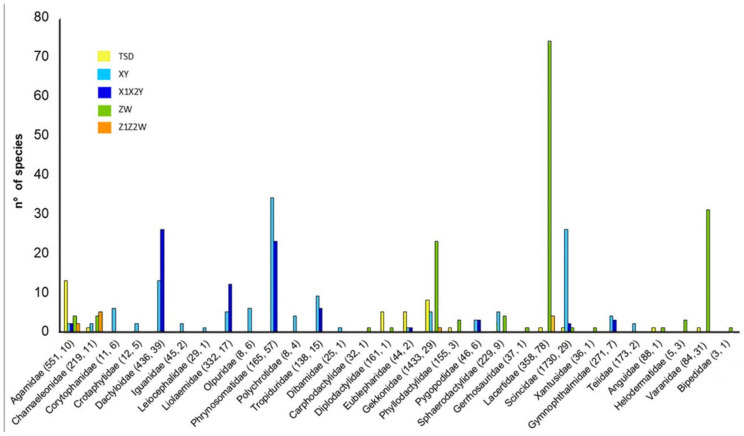
Qualitative and quantitative distribution of TSD and different sex chromosome systems in lizard families. In parentheses: number of described species for each family, followed by number of species with known TSD or sex chromosome system.

The distribution of different sex chromosome systems within families is also peculiar (Figure 4). In general, while some families show a comparable number of species with different sex determination systems, most of them are characterized by a clear prevalence of either male of female heterogamety (Figure 4 and Supplementary Materials Table S1). Agamidae, Diplodactylidae and Eublepharidae are the only three lizard families showing a predominance of TSD, which is also relatively well represented in Gekkonidae and occurs in only one or two species in other eight families (Figure 4 and Supplementary Materials Table S1).

Considering only groups with more than one record, male heterogamety is quantitatively predominant in 13 lizard families (XY + X_1_X_2_Y), while female heterogamety (ZW + Z_1_Z_2_W) is numerically more represented in seven families (Figure 4 and Supplementary Materials Table S1).

However, known sex chromosome systems are unevenly distributed among different lizard families. In particular, most of the available data concern eight families (Dactyloidae, Liolaemidae, Phrynosomatidae, Tropiduridae, Gekkonidae, Lacertidae, Scincidae and Varanidae), which, altogether, include 292 known sex chromosome system (78% of the total available data) (Supplementary Materials Table S1).

Most of the available data on male heterogamety come from Dactyloidae, Liolaemidae, Phrynosomatidae and Scincidae (Figure 4 and Supplementary Materials Table S1). The first three families also show a significant propensity for inter-chromosomal rearrangements involving the heterogametic chromosome and include most of the known lizard species with a X_1_X_2_Y system (66 out of 82 total records) (Figure 4 and Supplementary Materials Table S1). Similarly, most information on female heterogamety comes from Gekkonidae, Lacertidae and Varanidae, together including 128 out of 153 total records on ZW systems (Figure 4 and Supplementary Materials Table S1). On the other hand, sex chromosome systems in many families are known just from a handful of species, which nevertheless show an interesting variability of sex determination systems. For example, in Agamidae and Chamaeleonidae only 20 out of the 770 species so far described have a known sex determination system, but both families include species with either TSD, male and female heterogamety (Figure 4 and Supplementary Materials Table S1). It thus appears clear that, while recent studies and different methodological approaches greatly improved our knowledge on sex chromosome systems in lizards, a good portion of their systematic distribution remains unexplored. Multiple times, the amount and diversity of available data has been indicated as the main limiting factor in our knowledge of sex chromosome systems [153,154,155], and future analyses focused on completely unstudied and highly undersampled lizard families would help to greatly improve our understanding of mechanisms of sex chromosome evolution in vertebrates.

## 7. Cytogenetic and Molecular Methods for the Identification of Sex Chromosome Systems

A wide combination of cytogenetic and molecular techniques has been performed to detect genetic sex determination systems and/or identify sex chromosomes in lizards. These methods greatly vary in the quality of the results obtainable from different genomes and karyotypes and their successful application is dependent on the taxon studied.

Traditionally, sex chromosomes have been mostly identified in lizards with standard karyotyping, in species showing either dimensional and morphological differences between heteromorphic sex chromosomes or even multiple sex chromosome systems (e.g., References [56,156]). However, even if rare, heteromorphic autosomes are known in lizards (e.g., References [57,157]), and many species present homomorphic sex chromosomes which are undetectable without the application of more sophisticated techniques.

The advent of banding techniques provided useful tools in the study of sex chromosomes and helped to better understand their progressive steps of molecular and morphological diversification. C-banding selectively highlights constitutive heterochromatin and may identify heterochromatic sex chromosomes and different stages of their diversification (e.g., References [8,57,73]). On the other hand, sex chromosomes at initial diversification stages are not usually characterized by substantial differences in the amount of heterochromatin (e.g., References [87,88]) (see above and Figure 1) and can be therefore undetectable with banding techniques. Moreover, B chromosomes, which are mostly or completely composed of constitutive heterochromatin (e.g., References [158,159]), may appear very similar to degenerated heteromorphic sex chromosomes after C-banding. G-banding also finds useful applications in the study of sex chromosomes in lizards and has been employed in the identification of several heteromorphic sex chromosome systems (e.g., References [109,160]).

Molecular cytogenetics introduced a completely new range of techniques in the study of sex chromosome systems in vertebrates. For example, fluorescence in situ hybridization (FISH) using the sex-determining SRY gene finds consistent applications in mammals (e.g., References [161,162]). However, because sex-determining genes are not known in lizards, the identification of sex chromosomes with molecular cytogenetics involves the chromosomal mapping of other markers, including satellite repeats (which can preferentially accumulate on degenerated heteromorphic chromosomes), various sex-specific sequences (whose occurrence and localization may vary among different taxa) and painting techniques (e.g., with chromosome-specific probes) (e.g., References [71,99,104,163]).

Comparative genomic hybridization (CGH) has also been employed to identify sex chromosomes in lizards, but while it provided reliable results in some taxa, it was unable to detect sex chromosomes in other lineages (e.g., References [16,17,57,58]). Limits of CGH include its inability to detect undifferentiated, mostly pseudoautosomal sex chromosomes, as well as very small sex chromosomes, where hybridization signals may be hard to notice [11,58].

Two main molecular techniques, qPCR and RADseq, have the power to provide a qualitative/quantitative identification of sex-specific sequences, uncovering the occurrence of GSD and sex chromosome systems also in species with homomorphic sex chromosomes (e.g., References [13,76,128,164]). Moreover, qPCR is employed by using known genes, while RADseq can also identify the occurrence of sex chromosomes if genome assemblies are available from the same or a related species [165,166]. On the other hand, the detection of GSD does not correspond to the identification of the relative sex chromosome pair, which these methods are unable to provide unless coupled with molecular cytogenetics. In addition, in some cases the sex chromosomes found by cytogenetic methods were not detected by RADseq [157].

Single-chromosome sequencing (ChromSeq) is a further promising method in the study of sex chromosome system. By combining chromosome isolation (via microdissection or flow sorting), whole-genome amplification (WGA), hybridization and bioinformatic techniques ChromSeq has the power to the gap between cytogenetic and genomic studies [116,117,118]. So far, ChromSeq has been performed on just a handful of lizard species, but its application on a wider selection of taxa would help to uncover poorly study features of the lizard genome and karyotype [116,117,118].

In silico whole-genome subtraction (ISWGS), which has recently been performed on the skink *Acritoscincus duperreyi* [163], also has the potential to isolate sex-specific markers from heterogametic chromosomes (Y/W) and will probably help to open new perspectives in the study of sex chromosome evolution in squamate reptiles.

**Figure 5:**
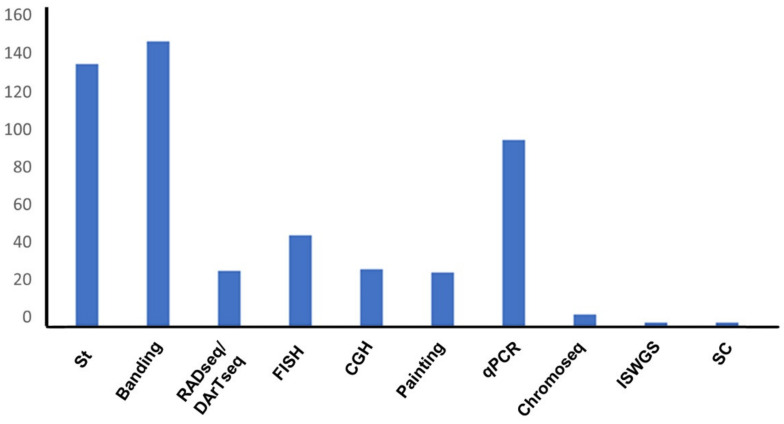
Experimental methods employed in the study of lizard sex chromosome systems; st = standard karyotyping; banding = banding techniques (C- and G-banding); painting = chromosome painting; ChromSeq = single-chromosome sequencing; ISWGS = in silico whole genome subtraction; SC = Synaptenemal Complex.

Most of the available data on sex chromosomes in lizards (Supplementary Materials Table S1) were obtained by using standard karyotyping (*n* = 137) and banding techniques (*n* = 137) (Figure 5), together covering more than 55% of the species with a known sex chromosome system. qPCR was also used on a good number of lizard species (*n* = 98), while RADseq and molecular cytogenetics have been applied in a relatively limited number of cases (Figure 5). Considering the strengths and limits of the currently available experimental techniques, future studies on lizard sex chromosome systems should preferably employ a combination of different cytogenetic and molecular methods, which would be able to unambiguously assess the occurrence of homomorphic and heteromorphic sex chromosome systems and better characterize their different diversification stages.

## 8. Conclusions

The study of sex chromosome evolution in lizards greatly improved our knowledge on the mechanisms and pathways of diversification of heteromorphic sex chromosomes, as well as on complex dynamics of sex reversal and sex chromosome turnover. Lizards are characterized by peculiar karyotype features, including a high variability in chromosome number, morphology and sex chromosome systems. Among vertebrates, their unparalleled diversity in sex determination includes a complex phylogenetic distribution of TSD, GSD and simple and multiple sex chromosome systems under either male or female heterogamety. Overall, male heterogamety is more common than female heterogamety in lizards, and while certain families show a clear prevalence of a particular sex chromosome system, most of them show a co-occurrence of different systems. However, although the increasing available information on lizard sex chromosomes started to provide a clearer general picture of the phylogenetic distribution of different systems, the available data still comprise less than 5% of the described species, leaving a good portion of their taxonomic diversity still unexplored. Furthermore, several aspects of sex determination in lizards remain completely unknown, including the sex-determining genes occurring in different families and sex chromosome systems. Future analyses should focus on the description of sex chromosome systems in undersampled or completely unstudied families, using a combination of different cytogenetic and molecular techniques. A better knowledge of the diversification of different sex determination and sex chromosome systems in lizards would represent a decisive contribution to our understanding of important evolutionary dynamics in vertebrates.

## Data Availability

The data considered in this study can be found in the manuscript and in Table S1.

## Supplementary Materials

The following are available online at https://www.mdpi.com/article/10.3390/genes12091341/s1. Table S1: Known sex chromosome systems in lizards and relative identification methods.
