# Supplementary material for: Lizards as Model Organisms of Sex Chromosome Evolution: What We Really Know from a Systematic Distribution of Available Data?
Source: Genes (Basel). 2021 Aug 28;12(9):1341. doi: 10.3390/genes12091341 (PMC8468487; doi:10.3390/genes12091341)
Supplement: Supplementary file 1 [file genes-12-01341-s001.zip › genes-1308172-supplementary.pdf]

Table 1. Karyotype description and known sex chromosome systems in lizards with relative identification methods. st = standard karyotyping; ISWGS = in silico whole genome subtraction SC = Synaptenemal Complex; nd = not determined; * cryptic. Systematics and taxonomy follow The Reptile Database [1].

Superfamily/Family	Species	Karyotype	Sex chrom.	Method	References
**Iguania**					
**Agamidae**					
	Calotes versicolor	2n=34♂ -33♀	Z_1Z_2W	st	[2]
	Ctenophorus fordi	2n=32	ZW	Banding, FISH	[3]
	Diporiphora nobbi	2n=32	ZW	FISH	[4]
	Pogona barbata	2n=32	ZW	FISH	[4,5]
	Pogona vitticeps	2n=32	X_1X_2Y	FISHq	[4,5]
	Sitana ponticeriana	2n=46♂ -45♀	Z_1Z_2W	st	[2]
	Tympanocryptis lineata	2n=32	ZW*	FISH	[6]
	Tympanocryptis pinguicolla	2n=32	ZW	DArTseq	[7]
	Uromastix ocellata	2n=36	XY	st	[8].
	Uromastix ornata	2n=36	XY	st	[8]
**Chamaeleonidae**	*Bradypodion ventrale*	2n=34♂ -35♀	Z_1Z_2W	st	[9]
	Chamaeleo calyptratus	2n=22	XY	RADseq	[10]
	Chamaeleo camaeleon	2n=22	XY	FISH, RADseq	[11]
	Furcifer bifidus	2n=23♀ -24♂	Z_1Z_2W	st, Banding CGH	[12]
	Furcifer lateralis	2n=24	ZW	st, Banding, CGH	[12]
	Furcifer oustaleti	2n=22	ZW	st, Banding, FISH	[13]
	Furcifer pardalis	2n=22♂ -21♀	Z_1Z_2W	st, Banding, FISH	[13]
	Furcifer verrucosus	2n=22♂ -21♀	Z_1Z_2W	st, Banding, CGH	[12]
	Furcifer wilsii	2n=27♀ -28♂	Z_1Z_2W	st, Banding, CGH	[12]
	Rhampholeon temporalis	2n=22	ZW (putative)	Banding, FISH	[14]
	Trioceros johnstoni	2n=36	ZW (putative)	Banding, FISH	[14]
**Corytophanidae**	*Basiliscus plumifrons*	2n=36	XY	RADseq	[15,16]
	Basiliscus vittatus	2n=36	XY	RADseq, Chromoseq	[16,17]
	Corytophanes cristatus	2n=36	XY	RADseq	[16]
	Corytophanes hernandesii	2n=36	XY	Chromoseq	[17]
	Laemanctus longipes	2n=36	XY	RADseq	[16]
	Laemanctus serratus	2n=36	XY	RADseq	[16]
**Crotaphytidae**					
	Crotaphytus collaris	2n=36	XY	qPCR	[18]
	Crotaphytus insularis	nd	XY*	qPCR	[16]

**Dactyloidae**	*Anolis acutus*	2n=34	X_1X_2Y	st	[19]
	Anolis allisoni	2n=36	XY	painting	[20]
	Anolis bartschi	2n=30	XY	painting	[20]
	Anolis bimaculatus	2n=29♂ -30♀	X_1X_2Y	st	[21]
	Anolis biporcatus (*Norops biporcatus*)	2n=29♂ -30♀	X_1X_2Y	st	[22]
	Anolis brevirostris	2n=33♂ -34♀	X_1X_2Y	st	[23]
	Anolis carolinensis	2n=36	XY	qPCR, painting, ChromoSeq	[20,24,25,26]
	Anolis chlorocyanus	2n=30	XY	qPCR	[27,28]
	Anolis conspersus	2n=30	XY	st	[29]
	Anolis cooki	2n=29♂ -30♀	X_1X_2Y	st	[30]
	Anolis cristatellus wileyae	2n=28♀ -27♂	X_1X_2Y	st	[30]
	Anolis cybotes	2n=36	XY	painting	[20]
	Anolis desechensis	2n=27♂ -28♀	X_1X_2Y	st	[23]
	Anolis distichus	2n=34♀ -33♂	X_1X_2Y	st, FISH	[19]
	Anolis equestris	2n=36	XY	painting	[22]
	Anolis evermanni	2n=26	XY	st	[19]
	Anolis ferreus	2n=29♂ -30♀	X_1X_2Y	st	[19]
	Anolis gingivinus	2n=29♂ -30♀	X_1X_2Y	st	[19]
	Anolis grahami	2n= 30	XY	FISH	[28]
	Anolis gundlachi	2n=29♂ -30♀	X_1X_2Y	st	[30]
	Anolis krugi	2n=29♂ -30♀	X_1X_2Y	st	[19]
	Anolis leachii	2n=29♂ -30♀	X_1X_2Y	st	[19]
	Anolis lineatopus (*Norops lineatopus*)	2n= 30	XY	FISH	[28,31]
	Anolis lividus	2n=29♂ -30♀	X_1X_2Y	st	[19]
	Anolis marmoratus	2n=29♂ -30♀	X_1X_2Y	st	[19]
	Anolis monensis	2n=29♂ -30♀	X_1X_2Y	st	[32]
	Anolis nubilus	2n=29♂ -30♀	X_1X_2Y	st	[32]
	Anolis oculatus	2n=32♀ -31♂	X_1X_2Y	st	[33]
	Anolis onca (*Norops onca*)	2n=30	XY	st	[31]
	Anolis pogus (*Ctnenonotus pogus*)	2n=29♂ -30♀	X_1X_2Y	Painting, Chromoseq	[20,34]
	Anolis poncensis	2n=29♂ -30♀	X_1X_2Y	st	[19,9]
	Anolis pulchellus	2n=29♂ -30♀	X_1X_2Y	st	[30,9]
	Anolis sabanus (*Ctenonotus sabanus*)	2n=29♂ -30♀	X_1X_2Y	st, painting, Chromosoeq	[19,9,20,34]
	Anolis sagrei (*Norops sagrei*)	2n=30	XY	st, painting	[35,9,20]

	Anolis scriptus leucophaeus	2n=28♀ -27♂	X_1X_2Y	st	[9,30]
	Anolis stratulus	2n=29♂ -30♀	X_1X_2Y	st	[9,19]
	Anolis uniformis	2n=29♂ -30♀	X_1X_2Y	st	[36]
	Anolis valencienni (*Norops valencienni*)	2n=30	XY	painting	[20]
	Anolis wattsi	2n=29♂ -30♀	X_1X_2Y	st	[19]
**Hoplocercidae**	no data				
**Iguanidae**	*Cyclura nubila*	nd	XY*	qPCR	[15]
	Iguana iguana	2n=36	XY	qPCR	[15,27]
**Leiocephalidae**	*Leiocephalus carinatus*	2n=34	XY	qPCR	[15,37]
**Leiosauridae**	no data available				
**Liolaemidae**	*Liolaemus lutzae*	2n=34	XY	st, Banding	[38]
	Liolaemus occipitalis	2n=34	XY	st, Banding	[38]
	Liolaemus wiegmanni	2n=34	XY	st, Banding	[38]
	Phymaturus antofagastensis	2n=27♂ -28♀	X_1X_2Y	st	[39]
	Phymaturus denotatus	2n=27♂ -28♀	X_1X_2Y	st, Banding	[40]
	Phymaturus dorsimaculatus	2n=35♂ -36♀	X_1X_2Y	st	[41]
	Phymaturus indistinctus	2n=42	XY	st	[42]
	Phymaturus laurenti	2n=27♂ -28♀	X_1X_2Y	st, Banding	[40]
	Phymaturus maulense (*P. palluma*)	2n=35♂ -36♀	X_1X_2Y	st	[43]
	Phymaturus palluma	2n=27♂ -28♀	X_1X_2Y	st, Banding	[40]
	Phymaturus palluma	2n=35♂ -36♀	X_1X_2Y	st, Banding	[40,43]
	Phymaturus patagonicus	2n=42	XY	st	[42]
	Phymaturus payuniae	2n=42	X_1X_2Y	st	[39]
	Phymaturus punae	2n=29♂ -30♀	X_1X_2Y	st	[42]
	Phymaturus roigorum	2n=27♂ -28♀	X_1X_2Y	st, Banding	[40]
	Phymaturus sp.	2n=27♂ -28♀	X_1X_2Y	st, Banding	[40]
	Phymaturus williamsi	2n=27♂ -28♀	X_1X_2Y	st, Banding	[40]
**Opluridae**	*Chalarodon madagascariensis*	2n=36	XY	qPCR, CGH	[15,44]
	Oplurus cuvieri	2n=36	XY	CGH	[44]

	Oplurus cyclurus	2n=36	XY	CGH	[44]
	Oplurus fierinensis	2n=36	XY	qPCR, CGH	[15,44]
	Oplurus grandidieri	2n=36	XY	CGH	[44]
	Oplurus quadrimaculatus	2n=36	XY	CGH	[44]
**Phrynosomatidae**	*Petrosaurus thalassinus*	nd	XY	qPCR	[15,24]
	Sceloporus aeneus	2n=24	XY	st	[45]
	Sceloporus angustus	2n=34	XY	st	[46]
	Sceloporus asper	2n=32♀ -31♂	X_1X_2Y	st	[45]
	Sceloporus bulleri	2n=32♀ -31♂	X_1X_2Y	st	[45]
	Sceloporus chrysostictus	2n=34	XY	st	[47]
	Sceloporus clarkii	2n=40	XY	st	[48]
	Sceloporus clarkii	2n=40♀ -39♂	X_1X_2Y	st	[46]
	Sceloporus couchii	2n=34	XY	st	[49]
	Sceloporus cozumelae	2n=34	XY	st	[49]
	Sceloporus cyanogenys	2n=32♀ -31♂	X_1X_2Y	st	[50]
	Sceloporus dugesii	2n=32♀ -31♂	X_1X_2Y	st	[45]
	Sceloporus gadoviae	2n=34	XY	st	[52]
	Sceloporus goldmani	2n=24	XY	st	[45]
	Sceloporus graciosus	2n=30	XY	SC	[53]
	Sceloporus grammicus	2n=32♀ -31♂	X_1X_2Y	st	[45]
	Sceloporus grammicus	2n=34♀ -33♂	X_1X_2Y	st	[54]
	Sceloporus grammicus	2n=36♀ -35♂	X_1X_2Y	st	[54]
	Sceloporus heterolepis	2n=32♀ -31♂	XY	st	[45]
	Sceloporus hunsakeri	2n=34	XY	st	[55]
	Sceloporus jalapae	2n=34	XY	st	[45]
	Sceloporus jarrovii	2n=32♀ -31♂	X_1X_2Y	st	[56]
	Sceloporus licki	2n=34	XY	st	[45]
	Sceloporus lundelli	2n=22	XY	st	[48]
	Sceloporus macdougalli	2n=32♀ -31♂	X_1X_2Y	st	[57]
	Sceloporus maculosus	2n=34♀ -33♂	X_1X_2Y	st	[46]
	Sceloporus malachiticus	2n=22	XY	qPCR, Banding, Chromoseq	[24,58]
	Sceloporus megalepidurus	2n=32♀ -31♂	X_1X_2Y	st	[45]
	Sceloporus merriami	2n=46	XY	st	[47]
	Sceloporus mucronatus	2n=32♀ -31♂	X_1X_2Y	st	[45]
	Sceloporus nelsoni	2n=34	XY	st	[52]
	Sceloporus ochoterenae	2n=34	XY	st	[49]
	Sceloporus orcutti	2n=34	XY	st	[48]
	Sceloporus ornatus	2n=32♀ -31♂	X_1X_2Y	st	[45]
	Sceloporus palaciosi	2n=34♀ -33♂	X_1X_2Y	st	[59]

	Sceloporus parvus	2n=34	XY	st	[45]
	Sceloporus poinsettii	2n=32♀ -31♂	X_1X_2Y	st	[60]
	Sceloporus pyrocephalus	2n=34	XY	st	[52]
	Sceloporus scalaris	2n=24	XY	st	[45]
	Sceloporus serrifer	2n=32♀ -31♂	X_1X_2Y	st	[45]
	Sceloporus shannonorum	2n=32♀ -31♂	X_1X_2Y	st	[45,51]
	Sceloporus siniferus	2n=34	XY	st	[45]
	Sceloporus smaragdinus	2n=22	XY	st	[61]
	Sceloporus teapensis	2n=34	XY	st	[49]
	Sceloporus torquatus	2n=32♀ -31♂	X_1X_2Y	st	[45]
	Sceloporus undulatus	2n=22	XY	st, SC	[62]
	Sceloporus utiformis	2n=34	XY	st	[47]
	Sceloporus variabilis	2n=34	XY	st, qPCR	[14,45]
	Sceloporus zosteromus (S. *rufidorsum*)	2n=30♀ -29♂	X_1X_2Y	st	[45]
	Uma inornata	2n=34	X_1X_2Y	st	[27]
	Uta nolascensis	2n=34	XY	st	[63]
	Uta palmeri	2n=34	XY	st	[63]
	Uta squamata	2n=34	XY	st	[62]
	Uta stansburiana	2n=34	XY	st	[63]
**Polychrotidae**	*Polychrus acutirostris*	2n=19♀ -20♀	X_1X_2Y	st	[64]
	Polychrus femoralis	2n=25♂ -26♀	X_1X_2Y	st	[65]
	Polychrus marmoratus	2n=29♂ -30♀	X_1X_2Y	st	[27]
	Polychrus peruvianus	2n=27♂ -28♀	X_1X_2Y	st	[65]
**Tropiduridae**	*Eurolophosaurus amathites* (*Tropidurus amathites*)	2n=36♀ -35♂	X_1X_2Y	Banding	[66]
	Tropidurus cocorobensis	2n=36	XY	Banding	[67,68]
	Eurolophosaurus divaricatus (*Tropidurus divaricatus*)	2n=36♀ -35♂	X_1X_2Y	Banding	[66]
	Tropidurus erythrocephalus	2n=36	XY	Banding	[67]
	Tropidurus etheridgei	2n=36	XY	Banding	[67,68]
	Tropidurus hispidus	2n=36	XY	Banding; qPCR	[68]
	Tropidurus hygomi	2n=36	XY	Banding	[67,68]
	Tropidurus itambere	2n=35♂ -36♀	X_1X_2Y	Banding	[67,68]
	Tropidurus montanus	2n=36	XY	Banding	[67]
	Tropidurus mucujensis,	2n=36	XY	Banding	[67]

	Eurolophosaurus nanuzae (*Tropidurus nanuzae*)	2n=35♂ -36♀	X_1X_2Y	Banding	[69]
	Tropidurus oreadicus	2n=36	XY	Banding	[67]
	Tropidurus psammonastes	2n=35♂ -36♀	X_1X_2Y	Banding	[66]
	Tropidurus torquatus	2n=35♂ -36♀	X_1X_2Y	Banding	[66]
	Uranoscodon superciliosus	nd	XY	qPCR	[15]
**Dibamia**					
**Dibamidae**	*Dibamus novaeguineae*	2n=36	XY	st	[70]
**Gekkota**					
**Carphodactylidae**	*Underwoodisaurus milii*	2n=38	ZW	Banding, CGH	[71]
**Diplodactylidae**	*Correlophus ciliatus (Rachodactylus ciliatus)*	2n=38	ZW	RADseq, painting	[72,73]
**Eublepharidae**	*Coleonyx elegans*	2n=31♂ -32♀	X_1X_2Y	st, painting, qPCR	[74,75]
	Coleonyx mitratus	nd	XY*	st, painting, qPCR	[75]
**Gekkonidae**	*Christinus marmoratus*	2n=36	ZW	Banding, RADseq	[75,72]
	Christinus m. marmoratus	2n=32	ZW	st, painting	[76,77]
	Cyrtodactylus chaunghanakwaensis	nd	XY	RADseq	[78]
	Cyrtodactylus pharbaungensis	nd	ZW	RADseq	[79]
	Cyrtodactylus pubisulcus	2n=42	ZW	Banding	[80]
	Cyrtopodion scabrum	2n=40	ZW	Banding	[81]
	Dixonius siamensis	2n=42	ZW	Banding	[82]
	Gehyra australis	2n=40	ZW	Banding	[83]
	Gehyra mutilata	nd	ZW	RADseq	[72]
	Gehyra nana	2n=44	ZW	Banding	[84]
	Gehyra purpurascens	2n=40	ZW	Banding	[85]
	Gekko gecko	2n=38	XY	Banding	[86]
	Gekko hokouensis	2n=38	ZW	Banding, painting	[77,87]
	Gekko japonicus	2n=38	XY	st	[88]
	Hemidactylus frenatus	2n=40	ZW	RADseq	[72,89]
	Hemidactylus -mabouia	2n=42	XY	RADseq	[72,90]
	Hemidactylus platyurus	2n=46	ZW?	Banding, painting	[91]
	Hemidactylus turcicus	2n=44	XY	RADseq	[72,92]
	Hemidactylus vietnamensis	3n = 60	ZW	st	[93,78]
	Heteronotia binoei	2n=42	ZW	Banding, RADseq	[72,94]
	Heteronotia binoei	3n=63	ZW	Banding	[94]

	Heteronotia sp.	2n=42	ZW	Banding	[95]
	~~*Lepidactylus*~~			Banding	[96]
	Lepidodactylus lugubris	2n=44	ZW		
	Lygodactylus picturatus	2n=39-41	0W*	Banding	[97]
	Paroedura cf. gracilis	2n=36♂ -35♀	Z_1Z_2W	st	[98]
	Paroedura lohatsara	2n=36	ZW	Banding, painting	[99]
	Paroedura masobe	2n=36	ZW	Banding, painting	[99]
	Paroedura karstophila	2n=36	ZW	Banding, painting	[99]
	Paroedura oviceps	2n=36	ZW	Banding, painting	[99]
	Paroedura stumpffi	2n=36	ZW	Banding, painting	[99]
**Phyllodactylidae**	*Phyllodactylus cf. lanei*	2n=34	ZW	Banding	[100]
	Phyllodactylus wirshingi	nd	ZW*	RADseq	[101]
	Thecadactylus rapicauda	2n=42	ZW	Banding, RADseq	[72,102]
**Pygopodidae**	*Aprasia parapulchella*	2n=42♀ -41♂	X_1X_2Y	CGH, qPCR	[103,104]
	Delma inornata	2n=36	XY	st, qPCR	[105,104]
	Delma butleri	?	XY	RADseq	[71]
	Lialis burtonis	2n=34♀ -33♂	X_1X_2Y	st, FISH, qPCR	[104,106,107]
	Lialis jicari	2n=42♀ -41♂	X_1X_2Y	St, FISH,	[104,107]
	Pygopus nigriceps	2n=38	XY	qPCR	[104,108]
**Sphaerodactylidae**	*Aristelliger barbouri*	nd	ZW*	RADseq	[109]
	Aristelliger expectatus	30	ZW	FISH, RADseq	[71,109]
	Aristelliger lar	nd	ZW*	RADseq	[109]
	Aristelliger praesignis	nd	ZW*	RADseq	[109]
	Euleptes europaea	2n=42	XY	FISH	[110]
	Gonatodes ceciliae	2n=22	XY	st	[111]
	Gonatodes humeralis	2n=32	XY	RADseq	[112,113]
	Sphaerodactylus macrolepis	nd	XY*	RADseq	[72]
	Sphaerodactylus nicholsi	nd	XY*	RADseq	[72]
**Scincomorpha**					
**Cordylidae**	no data available				
**Gerrhosauridae**	*Tracheloptychus petersi*	2n=34	ZW	CGH	[114]
**Lacertidae**	*Acanthodactylus boskianus*	2n=38	ZW*	qPCR	[115,116,117]

Acanthodactylus erythrurus	2n=38	ZW	Banding	[118]
Acanthodactylus erythrurus (*A. lineomaculatus*)	2n=38	ZW	Banding	[119]
Acanthodactylus schreiberi	2n=38	ZW	Banding, qPCR	[117,120]
Algyroides moreoticus	2n=38	ZW	Banding, qPCR	[117,121]
Algyroides nigropunctatus	2n=38	ZW	Banding, qPCR	[117,121]
Anatololacerta pelasgiana (*A. oertzeni*)	nd	ZW*	qPCR	[117]
Apathya cappadocica	nd	ZW*	qPCR	[117]
Atlantalacerta andreanskyi (*Lacerta andeanskyi*)	2n=38	ZW	qPCR	[117,122]
Darevskia armeniaca	2n=38	ZW	Banding	[123]
Darevskia dahli	2n=38	ZW	Banding, qPCR	[117,124]
Darevskia mixta	2n=38	ZW	Banding,	[124]
Darevskia raddei	2n=36	ZW	qPCR	[117,125]
Darevskia raddei nairensis	2n=36	ZW	FISH	[126,127]
Darevskia rardei x *portschinskii*	3n=57	ZW	qPCR	[117]
Darevskia rostombekowi	2n=36	ZW	Banding	[128]
Darevskia unisexualis	2n=38	ZW	Banding, FISH	[127,128]
Darevskia unisexualis x raddei	3n=57	ZW	Banding	[126,128]
Darevskia valentini	2n=36	ZW	Banding, FISH	[127,128]
Darevskia valentini	2n=38	ZW	st,	[127,129]
Dinarolacerta mosorensis	2n=38	ZW	St, Banding	[130]
Eremias arguta	2n=38	ZW	Banding, FISH	[129,131]
Eremias grammica	2n=38	ZW	Banding	[132]
Eremias multiocellata	2n=38	ZW	st, FISH, CGH	[133]
Eremias velox	2n=38	ZW	Banding, qPCR	[117,134,135]
Gallotia galloti	2n=40	ZW	Banding, FISH, qPCR	[116,117,120,136]
Gallotia stehlini	2n=40	ZW	qPCR	[117,137]
Gastropholis prasina	2n=38	ZW	Banding, FISH	[120]
Heliobolus lugubris	2n=38	ZW	Banding	[138]
Hellenolacerta graeca (Lacerta graeca)	2n=38	ZW	Banding	[121]
Holaspis guentheri	nd	ZW*	qPCR	[116,117]
Iberolacerta cyreni (*Lacerta cyreni*)	2n=36	ZW	Banding	[139,140]

Iberolacerta aurelioi (*Lacerta aurelioli*)	2n=26♂ -25♀	Z_1Z_2W	Banding	[139,140]
Iberolacerta bonnali (*Lacerta bonnali*)	2n=24♂ -23♀	Z_1Z_2W	Banding	[139,140]
Iberolacerta galani	2n=36	ZW	st, Banding, FISH	[140]
Iberolacerta horvathi *(Lacerta horvathi)*	2n=36	ZW	Banding, qPCR	[117,141]
Iberolacerta martinezricai	2n=36	ZW	Banding	[140]
Iberolacerta monticola	2n=36	ZW	Banding, FISH, qPCR	[117,139,140,142]
Iranolacerta brandtii (*Lacerta brandtii*)	2n=38	ZW	qPCR	[117,143]
Lacerta agilis	2n=38	ZW	Banding, qPCR, FISH	[116,117,121,144]
Lacerta bilineata	2n=38	ZW	Banding, qPCR	[116,117,120,136]
Lacerta media	2n=38	ZW	Banding, qPCR	[116,117,120,145]
Lacerta schreiberi	2n=38	ZW	Banding, FISH	[117,120,140,146]
Lacerta strigata	2n=38	ZW	Banding, FISH	[120,135,147]
Lacerta trilineata	2n=38	ZW	Banding, FISH	[117,120,148]
Lacerta viridis	2n=38	ZW	Banding	[117,121]
Latastia longicaudata	2n=38	ZW	Banding; FISH, qPCR	[117,120,149]
Meroles cuneirostris	2n=38	ZW	Banding	[118]
Meroles squamulosus	nd	ZW*	qPCR	[117]
Mesalina olivieri	2n=38	ZW	Banding, qQPR	[117,148]
Mesalina guttulata	2n=38	ZW	Banding, qPCR	[117,148]
Nucras intertexta	nd	ZW*	qPCR	[117]
Nucras taeniolata	nd	ZW*	qPCR	[117]
Omanosaura jayakari (*Lacerta jayakari*)	2n=38	ZW	Banding	[150]
Ophisops elegans	2n=38	ZW	Banding	[151]
Pedioplanis namaquensis	2n=38	ZW	Banding	[138]
Pedioplanis lineoocellata	nd	ZW*	qPCR	[117]
Phoenicolacerta kulzeri (*Lacerta kulzeri*)	2n=38	ZW	Banding	[117,152]
Phoenicolacerta laevis (*Lacerta laevis*)	2n=38	ZW	Banding	[116,152]
Phenicolacerta troodoca (*Lacerta troodoca*)	2n=38	ZW	Banding, FISH	[117, 120]
Podarcis bocagei	2n=38	ZW	qPCR	[117,153]
Podarcis hispanicus	2n=38	ZW	Banding, qPCR	[117,154]
Podarcis melisellensis	2n=38	ZW	st, Banding	[118,155]
Podarcis muralis	2n=38	ZW	qPCR	[116, 117,156]
Podarcis peloponnesiacus	2n=38	ZW	qPCR	[116, 117,146]

	Podarcis pityusensis	2n=38	ZW	qPCR	[117,157]
	Podarcis siculus	2n=38	ZW	Banding, FISH, qPCR	[117,120, 121]
	Podarcis tauricus	2n=38	Z_1Z2W	st, qPCR	[116,117,158]
	Podarcis waglerianus	2n=38	ZW	Banding	[140,159]
	Psammodromus algirus	2n=40	ZW	st, Banding, qPCR	[118,117155]
	Psammodromus hispanicus	2n=38	ZW	qPCR	[117,160]
	Scelarcis perspicillata (*Lacerta perspicillata*)	2n=38	ZW	qPCR	[117,161]
	Takydromus dorsalis	2n=38	ZW	Banding, FISH	[120]
	Takydromus sexlineatus	2n=38-42	ZW	Banding, qPCR	[116,117,120,162,]
	Teira dugesii (*Lacerta dugesii*)	2n=38	ZW	Banding, qPCR	[117,118]
	Timon lepidus (*Lacerta lepida*)	2n=36	ZW	Banding, FISH, qPCR	[116,117,120,163]
	Timon tangitanus	2n=36	ZW	Banding; FISH, qPCR	[117,120]
	Vhembelacerta rupicola	nd	ZW	qPCR	[117]
	Zootoca vivipara carniolica	2n=36	ZW	Banding	[164]
	Zootoca vivipara vivipara	2n=36♂ -35♀	Z_1Z_2W	Banding, qPCR	[116,117,166]
	Zootoca vivipara pannonica	2n=36♂ -35♀	Z_1Z_2W	Banding	[166]
**Scincidae**	*Ablepharus deserti*	2n=30	XY	st	[167]
	Ablepharus pannonicus	2n=28	XY	st	[167]
	Acritoscincus duperreyi	nd	XY*	ISWGS	[168]
	Asymblepharus alaicus	2n=30	XY	st	[167]
	Carinascincus ocellatus	2n=30	XY	FISH	[169]
	Chalcides viridanus	2n=28	XY	qPCR	[170,171]
	Corucia zebrata	2n=32	XY	Banding, qPCR	[171,172]
	Cyclodomorphus gerrardii (*Hemispheridion gerrardii*)	2n=32	XY*	qPCR	[171,172]
	Dasia olivacea	2n=32	XY	qPCR	[171,173]
	Emoia nigra	nd	XY	qPCR	[171]
	Eulamprus heatwolei	2n=30	XY	FISH, qPCR, ISWGS	[169,171,174]
	Eutropis multifasciata	nd	XY*	qPCR	[171]
	Lampropholis sp. C	2n=30	XY	Banding	[172]
	Liopholis whitii	2n=32	XY	FISH	[169]
	Mabuya mabouya mabouya	2n=30♀ -31♂	X_1X_2Y	st	[175]
	Oligosoma oliveri (*Cyclodina olivieri*)	2n=30	XY	st	[176]

	Pseudemoia entrecasteauxii	2n=30	XY	banding	[177]
	Saproscincus czechurai	2n=30	XY	Banding	[172]
	Scincella assata	2n=28	XY	banding	[178]
	Scincella cherriei	2n=30	XY	st, banding	[178]
	Scincella lateralis	2n=30♀ -29♂	$X_1X_2Y_2$	st	[179]
	Scincella melanosticta	2n=30	ZW	st	[180]
	Scincopus fasciatus	nd	XY*	qPCR	[171]
	Scincus scincus	2n=32	XY	qPCR	[156,171]
	Tiliqua gigas	2n=32	XY	Banding, qPCR	[171,172]
	Tiliqua nigrolutea	2n=32	XY	qPCR	[171,181]
	Trachylepis brevicollis	nd	XY*	qPCR	[171]
	Tribolonotus gracilis	2n=32	XY	Banding, qPCR	[171,172]
	Tropidophorus baconi	nd	XY*	CGH	[171]
**Xantusiidae**	*Xantusia henshawi*	40	ZW*	RADseq	[182]
**Gymnophthalmoidea**					
**Alopoglossidae**	no data available				
**Gymnophthalmidae**	*Calyptommatus leiolepis*	2n=58♀ -57♂	X_1X_2Y	Banding	[183]
	Calyptommatus nicterus	2n=58♀ -57♂	X_1X_2Y	Banding	[183]
	Calyptommatus sinebrachiatus	2n=58♀ -57♂	X_1X_2Y	Banding	[183]
	Gymnophthalmus pleii	2n=34	XY	Banding	[184]
	Micrablepharus atticolus	2n=50-53 (B chr.)	XY	Banding	[185]
	Micrablepharus maximiliani	2n=50-51 (B chr.)	XY	Banding	[185]
	Nothobachia ablephara	2n=62-64 (B chr.)	XY	Banding	[186]
**Teiidae**	*Aspidoscelis tigris* (*Cnemidophorus tigris*)	2n=46	XY	st	[187]
	Liopholis whitii	2n=32	XY	Banding, FISH	[169]
**Diploglossa**					
**Anguidae**	*Abronia lythrochila*	2n=30	ZW	Banding, CGH, qPCR	[188,189]
**Diploglossidae**	no data available				
**Xenosauridae**	no data available				

**Platynota**					
**Helodermatidae**	*Heloderma exasperatum*	2n=40	ZW	Banding, CGH, qPCR	[188,189]
	Heloderma horridum	2n=40	ZW	Banding, CGH, qPCR	[188,189]
	Heloderma suspectum	2n=36	ZW	Banding, CGH, qPCR	[188,190]
**Lanthanotidae**	no data available				
**Varanidae**	*Varanus acanthurus*	2n=40	ZW	Banding, painting, CGH, qPCR,	[191,188,192,193]
	Varanus albigularis	2n=40	ZW	Banding,	[194]
	Varanus beccarii	2n=40	ZW	Banding, CGH, qPCR, painting	[188,193]
	Varanus boehmei	2n=40	ZW	Bnding, painting	[193]
	Varanus cumingi	2n=40	ZW	Banding, CGH	[189]
	Varanus exanthematicus	2n=40	ZW	qPCR, RADseq	[182,195]
	Varanus gilleni	2n=40	ZW	qPCR	[194,188],
	Varanus glauerti	2n=40	ZW	painting, qPCR	[188,193]
	Varanus gouldii	2n=40	ZW	Banding, CGH	[192]
	Varanus indicus	2n=40	ZW	qPCR	[188,194]
	Varanus jobiensis	nd	ZW*	qPCR	[188]
	Varanus komodoensis	2n=40	ZW	Banding, FISH, qPCR	[188,194,195]
	Varanus kordensis	2n=40	ZW	Banidng, CGH, qPCR	[188,189]
	Varanus macraei	2n=40	ZW	Banding, painting	[193]
	Varanus melinus	nd	ZW*	qPCR	[188]
	Varanus mertensi	2n=40	ZW	FISH, qPCR	[188,193]
	Varanus niloticus	2n=40	ZW	Banding, CGH, FISH	[192,194]
	Varanus olivaceus	2n=40	ZW	Banding, FISH, qPCR	[188,189]
	Varanus pilbarensis	nd	ZW*	qPCR	[188]
	Varanus panoptes horni	2n=40	ZW	Banding, painting, qPCR	[188,193]
	Varanus prasinus	2n=40	ZW	Banding, paintingCR	[188,193]
	Varanus primordius	2n=40	ZW	Banding, FISH	[189]
	Varanus reisingeri	nd	ZW*	qPCR	[188]
	Varanus rosenbergi	2n=40	ZW	FISH, CGH	[192]
	Varanus salvadorii	2n=40	ZW	Banding, CGH, qPCR	[188,189]
	Varanus salvator komaini	2n=40	ZW	Banding, CGH, qPCR	[189]
	Varanus salvator macromaculatus	2n=40	ZW	Banding , FISH, CGH, qPCR	[188,192]
	Varanus tristis orientalis	2n=40	ZW	qPCR	[188,194]
	Varanus varius	2n=40	ZW	FISH, CGH, qPCR	[188,192]

**Shinisauroidea**					
**Shinisauridae**	no data available				
**Amphisbaenia**					
**Amphisbaenidae**	no data available				
**Bipedidae**	*Bipes tridactylus*	2n=46	ZW	st	[196]
**Blanidae**	no data available				
**Cadeidae**	no data available				
**Rhineuridae**	no data available				
**Trogonophidae**	no data available				

References

1. Uetz, P.; Freed, P.; Hošek, J. The Reptile Database **2020**, http://www.reptile-database.org, last accessed April 6, 2021.
2. Makino, S.; Asana, J.J. A sexual difference in the chromosomes of two species of agamid lizards. *Chromosoma* **1950**, *3*, 208-219.
3. Ezaz, T.; Quinn, A.E.; Sarre, S.D.; O'Meally, D.; Georges, A.; Graves, J.A.M. Molecular marker suggests rapid changes of sex-determining mechanisms in Australian dragon lizards. *Chromosome Res*. **2009**, *17*, 91-98; https://doi.org/10.1007/s10577-008-9019-5.
4. Matsubara, K.; O'Meally, D.; Sarre, S.D.; Georges, A.; Srikulnath, K.; Ezaz, T. ZW sex chromosomes in Australian dragon lizards (Agamidae) originated from a combination of duplication and translocation in the nucleolar organising region. *Genes* **2019**, *10*, 861; doi:10.3390/genes10110861.
5. Witten, G.J. Some Karyotypes of Australian Agamids (Reptilia: Lacertilia). *Aust. J. Zool*. **1983**, *31*, 533–540. https://doi.org/10.1071/ZO9830533
6. Alam, S.M.I.; Sarre, S.D.; Georges, A.; Ezaz, T. Karyotype characterisation of two australian dragon lizards (Squamata: Agamidae: Amphibolurinae) reveals subtle chromosomal rearrangements between related species with similar karyotypes. *Cytogenet. Genome Res*. **2020**, *160*, 610-623. https://doi.org/10.1159/000511344
7. Alam, S.M.I.; Georges, A.; Gleeson, D.; Sarre, S.D.; Ezaz, T. Sex-linked Markers in Australian grassland earless dragon *Tympanocryptis pinguicolla*, Mitchell, 1948. Combined Meeting of the Australian Society of Herpetologists and the Society for Research on Amphibians and Reptiles in New Zealand.Kindilan, Queensland, **2018**. DOI:10.13140/RG.2.2.19981.28648.
8. Utong, J.A.M.; Abukashawa, S.M.A. Characterization of the Agamid Lizard Genus *Uromastyx* from Eastern Sudan Based on Morphology, Karyotypes and Mitochondrial DNA. Asian *Herpetol. Res*. **2013**, *4*, 268–281; DOI: 10.3724/SP.J.1245.2013.00268.
9. Gordon, G.C. (Unpublished) in Olmo, E.; Signorino, G.G. Chromorep: A Reptile Chromosomes Database. **2005**. Available online: http://chromorep.univpm.it (accessed on 30 March 2021).
10. Nielsen, S.V.; Banks, J.L.R.; Diaz Jr, E.; Trainor P.A.; Gamble, T. Dynamic sex chromosomes in Old World chameleons (Squamata: Chamaeleonidae). *J. Evol. Biol.* **2018**, *31*, 484-490; DOI: 10.1111/jeb.13242.
11. Sidhom, M.; Said, K.; Chatti, N., Guarino, F.M.; Odierna, G.; Petraccioli, A.; Picariello, O.; Mezzasalma, M. Karyological characterization of the common chameleon (*Chamaeleo chamaeleon*) provides insights on the evolution and diversification of sex chromosomes in Chamaeleonidae. *Zoology* **2020**, *141*, 125738; https://doi.org/10.1016/j.zool.2019.125738.
12. Rovatsos, M.; Altmanová, M.; Augstenová, B.; Mazzoleni, S.; Velenský, P.; Kratochvíl, L. ZZ/ZW Sex Determination with Multiple Neo-Sex Chromosomes is Common in Madagascan Chameleons of the Genus *Furcifer* (Reptilia: Chamaeleonidae). *Genes* **2019**, *10*, 1020; doi:10.3390/genes10121020.
13. Rovatsos, M.; Pokorná, M.J.; Altmanová, M.; Kratochvíl, L. Female heterogamety in Madagascar chameleons (Squamata: Chamaeleonidae: *Furcifer*): dierentiation of sex and neo-sex chromosomes. *Sci. Rep.* **2015**, *5*, 13196; https://doi.org/10.1038/srep13196.
14. Rovatsos, M.; Altmanová, M.; Johnson Pokorná, M.; Velenský, P.; Sánchez Baca, A.; Kratochvíl, L. Evolution of karyotypes in chameleons. *Genes* **2017**, *8*, 382. https://doi.org/10.3390/genes8120382.
15. Rovatsos, M.; Pokorná, M.; Altmanová, M.; Kratochvíl, L. Cretaceous park of sex determination: sex chromosomes are conserved across iguanas. *Biol. Lett.* **2014**, *10*, 20131093. http://dx.doi.org/10.1098/rsbl.2013.1093
16. Nielsen, S.V.; Guzmán-Méndez, I.A.; Gamble, T.; Blumer, M.; Pinto, B.J.; Kratochvíl, L.; Rovatsos, M. Escaping the evolutionary trap? Sex chromosome turnover in basilisks and related lizards (Corytophanidae: Squamata). *Biol. Lett.* **2019**, *15*: 20190498; http://dx.doi.org/10.1098/rsbl.2019.0498.
17. Acosta, A.; Suárez-Varón, G.; Rodríguez-Miranda, L.A.; Lira-Noriega, A.; Aguilar-Gómez, D.; Gutiérrez-Mariscal, M.; Hernández-Gallegos, O.; Méndez-de-la-Cruz, F.; Cortez, D. Corytophanids replaced the pleurodont XY system with a new pair of XY chromosomes. *Genome Biol. Evol.* **2019**, *11*, 2666-2677. https://doi.org/10.1093/gbe/evz196.
18. Wiggins, J.M.; Santoyo-Brito, E.; Scales, J.B.; Fox, S.F. Gene dose indicates presence of sex chromosomes in collared lizards (C*rotaphytus collaris*), a species with temperature-influenced sex determination. *Herpetologica,* **2020**, *76*, 27-30. https://doi.org/10.1655/Herpetologica-D-19-00036.
19. Gorman, G.C; Atkins, L. The zoogeography of Lesser Antillean Anolis lizards-an analysis based upon chromosomes and lactic dehydrogenases. *Bull. Mus. Comp. Zool*. **1969**, *138*, 53–80.
20. Giovannotti, M.; Trifonov, V.A.; Paoletti, A.; Kichigin, G.; O'Brien, P.C.M.; 6, Kasai, F.; Giovagnoli, G.; Ng B.L.; Ruggeri, P.; Nisi Cerioni, P., Splendiani, A., Pereira, J.C.; Olmo, E.; Rens, W.; Caputo Barucchi, V., Ferguson-Smith, M.A. New insights into sex chromosome evolution in anole lizards (Reptilia, Dactyloidae). *Chromosoma* **2017**, *126*, 245-260; doi: 10.1007/s00412-016-0585-6.
21. Gorman, G. Interspecific karyotypic variation as a systematic character in the genus Anolis (Sauria: Iguanidae). *Nature*, 1**965**, *208*, 95–97.
22. Gorman, G.C.; Atkins, L. Chromosomal heteromorphism in some male lizards of the genus Anolis. *Amer. Naturalist*, **1966**, *100*, 579–583.
23. Webster T.B. in Brandley, M.C.; Wynn, A.; De Queiroz, K. Karyotype and Relationships of *Anolis desechensis*. *J. Herpetol*. **2006**, *40*, 136–139: DOI: 10.1670/98-05N.1.
24. Rovatsos, M.; Altmanová, M.; Pokorná, M.; Kratochvíl, L. Conserved Sex Chromosomes Across Adaptively Radiated *Anolis* Lizards. *Evolution* **2014**, *68*, 2079–2085. doi:10.1111/evo.12357.
25. Alföldi, J.; Di Palma, F.; Grabherr, M. et al. The genome of the green anole lizard and a comparative analysis with birds and mammals. *Nature* 2011, *477*, 587–591 https://doi.org/10.1038/nature10390.
26. Iannucci, A.; Makunin, A.I.; Lisachov, A.P.; Ciofi, C.; Stanyon, R.; Svartman, M.; Trifonov, V.A. Bridging the Gap between Vertebrate Cytogenetics and Genomics with Single-Chromosome Sequencing (ChromSeq). *Genes* **2021***, 12*, 124
27. Gorman, G.C.; Atkins, L.; Holzinger. T. New karyotypic data on 15 genera of lizards in the family Iguanidae, with a discussion of taxonomic and cytological implications. *Cytogenetics (Basel)* **1967**, *6*, 286–299. https://doi.org/10.1159/000129949
28. Gamble, T.; Geneva, A.J.; Glor, R.E., Zarkower, D. *Anolis* sex chromosomes are derived from a single ancestral pair. *Evolution* **2014**, *68,* 1027–1041. doi:10.1111/evo.12328.
29. Gorman, G.C., Atkins, L. Confirmation of an X-Y sex determining mechanism in lizards (Anolis). *Copeia*, **1968**, *1*: 159–160.
30. Gorman, G.C.; Thomas, R., Atkins, L. Intra- and interspecific chromosome variation in the lizard *Anolis cristatellus* and its closest relatives. *Breviora, Mus. Comp. Zool*., **1968**, *293*, 1-13.
31. Gorman, G.C. Chromosomes of three species of anoline lizards in the genera Anolia and Tropidodactylus. *Mammal. Chrom. Newsl*., **1969**, 10: 222–224.
32. Gorman, G.C.; Stamm, B. The Anolis Lizards of Mona, Redonda, and La Blanquilla: Chromosomes, Relationships, and Natural History Notes. *J. Herpetol*. **1975**, *9*, 197-205. https://doi.org/10.2307/1563037
33. Gorman, G.C.; Atkins, L. The relationships of Anolis of the roquet species group (Sauria: Iguanidae). II-Comparative chromosome cytology. *Syst. Zool*. **1967**, *16*:137-143.
34. Lisachov, A.P.; Makunin, A.I.; Giovannotti, M.; Pereira, J.C.; Druzhkova, A.S.; Caputo Barucchi, V.; Ferguson-Smith, M.A.; Trifonov, V.A. Genetic content of the neo-sex chromosomes in *Ctenonotus* and *Norops* (Squamata, Dactyloidae) and degeneration of the Y chromosome as revealed by high-throughput sequencing of individual chromosomes. *Cytogenet. Genome Res*. **2019**. *157*, 115-122.
35. De Smet, W.H.O. Description of the orcein stained karyotypes of 27 lizard species (Lacertilia, Reptilia) belonging to the families Iguanidae, Agamidae, Chameleontidae and Gekkonidae (Ascalabota). *Acta Zool. Pathol. Antverpiensia* **1981**, *76,* 35-72.
36. Castiglia, R.; Flores-Villela, O.A.; Bezerra, A.M.R.; Gornung, E.; Annesi, F.; Muñoz-Alonso, L.A.; Solano, E. Detection of cryptic diversity in lizards (Squamata) from two Biosphere Reserves in Mesoamerica. *Comp. Cytogen*. **2020**, *14*, 613-638.
37. Porter, C.A.; Crombie, R.I.; Baker, R.J. Karyotypes of five species of Cuban lizards. *Occas. Papers Mus., Texas Tech Univ*., **1989**, *130*, 1-6.

38. Bertolotto, C.E.V.; Rodrigues, M.T.; Skuk, G.; Yonenaga-Yassuda Y. Comparative cytogenetic analysis with differential staining in three species of *Liolaemus* (Squamata, Tropiduridae). *Hereditas* **1996,** *125*, 257-264; https://doi.org/10.1111/j.1601-5223.1996.00257.x.
39. Pereyra, E.A. 1991.Unpublished datum) in Grosso, J.R., Cardozo, D.; Baldo, D., Lobo, F. Multiple sex chromosome system and robertsonian rearrangements involved in the chromosome evolution of the *Phymaturus palluma* group (Iguania: Liolaemidae). *J. Herpetol*. **2017**, *51*, 154-160.
40. Grosso, J.R.; Cardozo, D.; Baldo, D.; Lobo, F. Multiple sex chromosome system and robertsonian rearrangements involved in the chromosome evolution of the *Phymaturus palluma* group (Iguania: Liolaemidae). *J. Herpetol*. **2017**, *51*, 154-160.
41. Morando, M.; Guerreiro, A.; Avila, L. J. **2001** (unpublished datum) in Grosso, J.R., Cardozo, D.; Baldo, D., Lobo, F. Multiple sex chromosome system and robertsonian rearrangements involved in the chromosome evolution of the *Phymaturus palluma* group (Iguania: Liolaemidae). *J. Herpetol*. **2017**, *51*, 154-160.
42. Morando, M. 2004 (unpublished datum) in Grosso, J.R., Cardozo, D.; Baldo, D., Lobo, F. Multiple sex chromosome system and robertsonian rearrangements involved in the chromosome evolution of the *Phymaturus palluma* group (Iguania: Liolaemidae). *J. Herpetol*. **2017**, *51*, 154-160.
43. Lamborot, M.; Navarro-Sua´ Rez, N. 1984. Karyotypes and sex determination in *Phymaturus palluma* Molina (Iguanidae). *Herpetologica*, **1984**, *40,* 258–264.
44 Altmanová, M.; Rovatsos, M.; Kratochvíl, L.; Johnson Pokorná, M. Minute Y chromosomes and karyotype evolution in Madagascan iguanas (Squamata: Iguania: Opluridae). *Biol. J. Lin. Soc.* **2016**, *118*, 618-633.
45. Hall, W.P. Comparative population cytogenetics, speciation, and evolution of the creviceusing species of *Sceloporus* (Sauria, Iguanidae). Ph.D. thesis, Harvard University, Cambridge, **1973.**
46. Leaché, A.D.; Sites Jr., J.W. Chromosome evolution and diversification in North American spiny lizards (Genus *Sceloporus*). *Cytogenet. Genome Res*. **2009**, *127*,166-181; DOI: 10.1159/000293285.
47.Cole, C. J. Karyotypes of the five monotypic species groups of lizards in the genus *Sceloporus*. *Amer. Mus. Novitates* **1971**, *2450*, 1-17.
48. Cole, C. J. Karyotypes and evolution of the spinosus group of lizards in the genus *Scelopo*rus. *Amer. Mus. Novitates* **1970**, *2431*, 1-47.
49. Cole, C. J. Karyotypes and Systematics of the Lizards in *variabilis*, *jalapae*, and *scalaris* Species Groups Genus *Sceloporus. Amer. Mus. Novitates* **1978**, *2653*, 1-13.
50. Olson, R.E. Taxonomic revision of the lizards *Sceloporus serrifer* and *cyanogenys* of the Gulf Coastal Plain. *Bull. Maryland Herpetol. Soc*. **1987**, *23*, 158-16750.
51. Sites Jr, J.W.; Archie, J.W.; Cole, C.J.; Villela, O.F. A review of phylogenetic hypotheses for lizards of the genus *Sceloporus* (Phrynosomatidae): implications for ecological and evolutionary studies. *Bul. Am. Mus. Nat. Hist*. **1992,** *213*, 1-110. http://hdl.handle.net/2246/908.
52.Cole, C. J. Karyotypes and relationships of the *pyrocephalus* group of lizards in the genus Sceloporus. *Herpetologica* **1971**, *27*, 1-8.
53. Reed, K.M.; Sudman, P.D.; Sites Jr., J.W.; Greenbaum I.F. Synaptonemal Complex Analysis of Sex Chromosomes in Two Species of *Sceloporus. Copeia* **1990***, 4,* 1122-1129. https://doi.org/10.2307/1446497
54. Sites, J.W. Jr. Chromosome evolution in the iguanid lizard *Sceloporus grammicus*. I. Chromo- some polymorphism. *Evolution* **1983**, *37,* 38-53.
55. Hall, W.P.; Smith H.M. Lizards of the *Sceloporus orcutti* complex of the Cape region of Baja California. *Breviora* **1979**, *452*, 1-26.
56. Lowe, C. H.; Wright, J. W. Chromosomes and karyotypes of cnemidophorine teiid lizards. *Mamm. Chrom. Newsletter* **1966**, *22*, 199-200.
57. Mendoza-Quijano, F.; Goynechea, I. The karyotype of *Sceloporus macdougallii* (Squamata: Prynosomtidae). *Rev. Esp. Herp*. **2004**, *18*, 75-78.
58. Lisachov, A.P.; Tishakova, K.V.; Romanenko, S.A.; Molodtseva, A.S.; Prokopov, Y.D.; Pereira, J.C.; Ferguson-Smith, M.A.; Borodin, P.M.; Trifonov, V.A. Whole-chromosome fusions in the karyotype evolution of Sceloporus (Iguania, Reptilia) are more frequent in sex chromosomes than autosomes. PTRS-B. https://doi.org/10.1098/rstb.2020.0099
59. Hall, W.P.; Selander, R.K. Hybridization of karyotypically differentiated populations in the *Sceloporus grammicus complex* (Iguanidae). *Evolution* **1973**, *27*, 226-242.
60. Cole, C.J.; Lowe, C.H.; Wright, J.W. 1967. Sex chromosomes in lizards. *Science*, **1967**, *155*, 1028-1029.
61. Goyenechea, I.; Mendoza-Quijano, F. Descripción del cariotipo de *Sceloporus smaragdinus* (Sauria: Phrynosomatidae). *Bol. Soc. Herp. Mex*. **1993**, *5*, 62-64.
62. Reed, K.M.; Sudman, P.D.; Sites Jr, J.W.; Greenbaum, I.F. Synaptonemal Complex Analysis of Sex Chromosomes in Two Species of *Sceloporus*. *Copeia* **1990**, *4*, 1122-1129. https://doi.org/10.2307/1446497.
63. Pennock, A.E.; Tinkle, D.W.; Shaw, M.W. Minute Y chromosome in the lizard genus *Uta* (family Iguanidae). *Cytogenetics* **1969**, *8*, 9-19.
64. Peccinini, D.; Frota-Pessoa, O.; Ferrari, I. Sex determination of the pseudo XO/XX type in the Brazilian lizard *Polychrus* sp. (Sauria, Iguanidae). *Caryologia* **1971**, *24*, 129-139.
65. Gorman, G.C.; Huey, R. B., Williams, E. E. Cytotaxonomic studies on some unusual iguanid lizards assigned to the genera *Chamaeleolis*, *Polychrus*, *Polychroides*, and *Phenacosaurus*, with behavioral notes. *Breviora* **1969**, *316*, 1-17.
66. Kasahara, S.; Yonenaga-Yassuda, Y.; Rodrigues, M.T. Karyotype and evolution of the *Tropidurus nanuzae* species group (Sauria, Iguanidae). *Rev. Bras. Genet*. **1987**, *10*, 185-197.
67. Kasahara, S.; Machado Pellegrino, K.C.; Rodrigues M.T.; Yonenaga-Yassuda. Y. Comparative cytogenetic studies of eleven species of the *Tropidurus torquatus* group (Sauria, Tropiduridae), with banding patterns. *Hereditas* **1996**, *125*, 37-46. https://doi.org/10.1111/j.1601-52.
68. Kasahara, S.; Yonenaga-Yassudya, Y.; Rodrigues, M.T. Geographical karyotypic variations and chromosome banding patterns in *Tropidurus hispidus* (Sauria, Iguanidae) from Brazil. - *Caryologi*a **1987**, **4**, 43-57. https://doi.org/10.1080/00087114.1987.10797808.
69. Kasahara, S.; Yonenaga-Yassudya Y.; Rodrigues, M.T. Karyotype and evolution of the *Trupidurus nanuzue* species group (Sauria, Iguanidae). *Rev. Brasil. Genet*. **1987**, *X*, 185-197
70. Cole, C. J.; Gans, C. The Karyotype of *Dibamus novaeguineae* (Squamata: Dibamidae). *Herpetologica* **1997**, *53*, 229-232.
71. Pokorná, M.; Rens, W.; Rovatsos, M.; Kratochvíl, L. A ZZ/ZW sex chromosome system in the thick-tailed gecko (*Underwoodisaurus milii*; Squamata: Gekkota: Carphodactylidae), a member of the ancient gecko lineage. *Cytogenet. Genome Res*. **2014**,*142*, 190-19. doi: 10.1159/000358847.
72. Gamble, T.; Coryell, J.; Ezaz, T., Lynch, J.; Scantlebury, D.P.; Zarkower, D. Restriction Site-Associated DNA Sequencing (RAD-seq) Reveals an Extraordinary Number of Transitions among Gecko Sex-Determining Systems. *Mol. Biol. Evol*. **2015**, *32*, 1296-1309. doi:10.1093/molbev/msv023.
73. Pokorná, M.; Giovannotti, M.; Kratochvíl, L.; Kasai. F.; Trifonov, V.A.; O'Brien, P.C.; Caputo, V.; Olmo, E.; Ferguson-Smith, M.A.; Rens. W. Strong conservation of the bird Z chromosome in reptilian genomes is revealed by comparative painting despite 275 million years divergence. *Chromosoma* **2011**, *120*, 455–468. DOI: 10.1007/s00412-011-0322-0
74. Pokorná, M.; Rábová, M.; Ráb, P.; Ferguson-Smith, M.A.; Rens, W.; Kratochvíl, L. Differentiation of sex chromosomes and karyotypic evolution in the eye-lid geckos (Squamata: Gekkota: Eublepharidae), a group with different modes of sex determination. *Chromosome Res*. **2010**, *18*, 809-820. DOI 10.1007/s10577-010-9154-7.
75 Pensabene, E.; Kratochvíl, L.; Rovatsos, M. Independent Evolution of Sex Chromosomes in Eublepharid Geckos, A Lineage with Environmental and Genotypic Sex Determination*. Life* **2020**, *10*, 342. doi:10.3390/life10120342.
76. King, M.; Rofe, R. Karyotypic variation in the Australian gekko *Phyllodactylus marmoratus* Gray*. Chromosoma,* **1976***, 54:*75-87.74.
77 Matsubara, K.; Gamble, T.; Matsuda, Y., Zarkower, D.; Sarre, S.D; Georges, A.; Marshall Graves, J.A.; Ezaz, T. Non-Homologous Sex Chromosomes in Two Geckos (Gekkonidae: Gekkota) with Female Heterogamety. Cytogenet. Genome Res. **2014**, *143*, 251-258. DOI: 10.1159/000366172.
78.Gamble, T. A Review of Sex Determining Mechanisms in Geckos (Gekkota: Squamata). *Sex Dev*. **2010**, *4*, 88-103; DOI: 10.1159/000289578.

79. Keating, S.E.; Blumer, M.; Grismer L.L.; Lin, A.; Nielsen, S.V.; Thura, M.K.; Wood Jr., P.L., Quah, E.S.H.; Gamble, T. Sex Chromosome Turnover in Bent-Toed Geckos (*Cyrtodactylus*). *Genes* **2021**, *12*, 116. https://doi.org/10.3390/genes12010116.
80. Ota, H.; Hikida, T.; Matsui, M.; Mori, A. Karyotypes of two species of the genus *Cyrtodactylus* (Squamata: Gekkonidae) from Sarawak, Malaysia. *Caryologia* **1992**, *45,* 43–49. DOI: 10.1080/00087114.1992.10797209
81. Moghanjoghi, S.M.; Ganjibakhsh, M.; Gohari, N.S.: Izadpanah, M.; Rahmati, H.; Elyasi, Z.; Mohebali, G.N.; Vakhshiteh, F.; Farzaneh, P. Establishment and characterization of rough-tailed gecko original tail cells. *Cytotechnology* **2018**, *70*, 1337-1347; https://doi.org/10.1007/s10616-018-0223-7.
82.Ota, H.; Hikida, T.; Nabhitabhata, J.; Panha, S. Cryptic taxonomic diversity in two broadly distributed lizards of Thailand (*Mabuya macularia* and *Dixonius siamensis*) as revealed by chromosomal investigations (Reptilia: Lacertilia). *Nat Hist J Chulalongkorn Univ*., **2001**, *1*, 1–7.
83. King, M. King M: The Evolution of Sex Chromosomes in Lizards, In *Evolution and Reproduction*, Calaly, J., Tyndale-Biscoe, H. Eds.; Australian Academy of Science, Canberra 1977, pp 55–60.
84. Moritz, C. The population biology of *Gehyra* (Gekkonidae): chromosome change and speciation*. Syst. Zool.* **1986**, *35*, 46-67.
85. King, M. Karyotypic evolution in *Gehyra* (Gekkonidae, Reptilia). IV. Chromosome change and speciation. *Genetica* **1984**, *64*, 101–114.
86. Soolender, E.; Schmid, M. XX/XY-Sex Chromosomes in Gekko gecko (Sauria, Reptilia). *Amphib.-Reptil.***, 1984***, 5*, 339-345
87. Kawai, A.; Ishijima, J.; Nishida, C.; Kosaka, A.; Ota, H.; Kohno, S.; Matsuda, Y. The ZW sex chromosomes of *Gekko hokouensis* (Gekkonidae, Squamata) represent highly conserved homology with those of avian species. *Chromosoma* **2009**, *118*, 43-51. https://doi.org/10.1007/s00412-008-0176-2.
88.Yoshida, M.; Itoh, M. Karyotype of the gecko, *Gekko japonicus . Chrom. Inf. Serv.* **1974***, 17*, 29–31.
89. King, M. A new chromosome form of *Hemidactylus frenatus* (duméril and bibron). *Herpetologica* **1978**, *34*, 216-218
90. McBee, K.; Bickham, J.W.; Dixon, J.R.; Male heterogamety and chromosomal variation in Caribbean geckos. *J. Herpetol.* **1987**, *21*:68–71.
91.Trifonov, V.A.; Giovannotti, M.; O'Brien, P.C.M.; Wallduck, M.; Lovell, F.; Rens, W.; Parise-Maltempi, P.P.; Caputo, V.; Ferguson-Smith, M.A. Chromosomal evolution in Gekkonidae. I. Chromosome painting between *Gekko* and *Hemidactylus* species reveals phylogenetic relationships within the group. *Chromosome Res*. **2011**, *19*, 843-855; DOI 10.1007/s10577-011-9241-4.
92. Werner, Y.L. Chromosome numbers of some male geckos (Reptilia: Gekkonoidea). Bull. Res. Counc. Israel, 1956, 5B, 319.
93. Darevsky, I.S.; Kupriyanova, L.A.; Roshchin. V.V. A new all-female triploid species of gecko and karyological data on the bisexual *Hemidactylus frenatus* from Vietnam. *J Herpetol.* 1984, *18,* 277–284.
94. Moritz, C. The origin and evolution of parthenogenesis in *Heteronotia binoei* (Gekkonidae) I. Chromosome banding studies. *Chromosoma* **1984**, *89*, 151–162. doi:10.1007/BF00292899
95. Moritz, C. Patterns and processes of sex chromosome evolution in Gekkonid lizards (Sauria: Reptilia). In Cytogenetics of amphibians and reptiles; Olmo, E., Ed,; Birkhäuser-Verlag Berlin, Germany, 1990, pp. 205–219.
96. Volobouev, V.; Pasteur, G. Presumptive sex chromosomes of a unisexual homomorphic species of lizards, *Lepidodactylus lugubris*. *Heredity*, **1988**, *60*, 463–467. doi:10.1038/hdy.1988.65)
97. Castiglia, R. First chromosomal analysis for the genus *Lygodactylus* (Gray, 1864): the karyotype of *L. picturatus* (, Squamata, Gekkonidae Gekkoninae). *Afr. J. Herpetol*. **2004**, *53*, 95-97; https://doi.org/10.1080/21564574.2004.9635502.
98. Aprea, G.; Andreone, F.; Fulgione, D.; Petraccioli, A.; Odierna, G. Chromosomal rearrangements occurred repeatedly and independently during species diversification in Malagasy geckos, genus *Paroedura*. *Afr. Zool*. **2013**, *48*, 96-108. https://doi.org/10.1080/15627020.2013.11407572.
99. Koubová, M.; Johnson Pokorná, M.; Rovatsos, M.; Farkačová, K.; Altmanová, M.; Kratochvíl, L. Sex determination in Madagascar geckos of the genus *Paroedura* (Squamata: Gekkonidae): are differentiated sex chromosomes indeed so evolutionary stable? *Chromosome Res*. **2014**, *22*, 441-452. doi: 10.1007/s10577-014-9430-z.
100. King, M. Chromosome change and speciation in lizards. In *Evolution and speciation*, Atchley, W.R., Woodruff, D., Eds.; Cambridge University Press, London, UK, **1981** pp. 262-285.
101. Nielsen, S.V.; Daza, J.D.; Pinto, B.J.; Gamble, T. ZZ/ZW Sex Chromosomes in the Endemic Puerto Rican Leaf-Toed Gecko (*Phyllodactylus wirshingi*). *Cytogenet. Genome Res*. **2019**, *157*, 89-97. doi: 10.1159/000496379.
102. Schmid, M.; Steinlein, C.; Haaf, T.; Mijares-Urrutia, A. Nascent ZW Sex Chromosomes in *Thecadactylus rapicauda* (Reptilia, Squamata, Phyllodactylidae). *Cytogenet. Genome Res*. **2014**, *143*, 259-267. https://doi.org/10.1159/000366212.
103. Matsubara, K.; Knopp, T.; Sarre, S.D.; Georges, A.; Ezaz, T. Karyotypic analysis and FISH mapping of microsatellite motifs reveal highly differentiated XX/XY sex chromosomes in the pink-tailed worm-lizard (*Aprasia parapulchella*, Pygopodidae, Squamata). *Mol. Cytogenet*. **2013**, *6*, 60. http://www.molecularcytogenetics.org/content/6/1/60.
104. Rovatsos, M.; Gamble, T.; Nielsen, S.V.; Georges, A.; Ezaz, T.; Kratochvíl, L. Do male and female heterogamety really differ in expression regulation? Lack of global dosage balance in pygopodid geckos. *Phil. Trans. R. Soc. B.*, **2021,** *376,* 20200102. doi.org/10.1098/rstb.2020.0102
105. King, M. Chromosomal and immunogenetic data: a new perspective on the origin of Australia's reptiles, In: Cytogenetics of Amphibians and Reptiles, Olmo, E., Ed.; Birkhäuser, Basel, Switzerland, 1990, pp. 153–180.
106. Gorman, G.C.; Gress, F. Sex chromosomes of a pygopodid lizard, *Lialis burtonis*. *Experientia*, **1970**, *26*, 206–207. doi: 10.1007/BF01895586.
107. Rovatsos, M.; Johnson Pokorná, M.; Altmanová, M.; Kratochvíl, L. Mixed-up sex chromosomes: identification of sex chromosomes in the $X_1X_1X_2X_2$ /X_1X_2Y system of the legless lizards of the genus *Lialis* (Squamata: Gekkota: Pygopodidae). *Cytogenet. Genome Res.* **2016**, *149*, 282-289. doi: 10.1159/000450734.
108. Rovatsos, M.; Gamble, T.; Nielsen, S.V.; Georges, A.; Ezaz, T.; Kratochvíl, L. Do male and female heterogamety really differ in expression regulation? Lack of global dosage balance in pygopodid geckos. *bioRxiv* **2020.** doi: https://doi.org/10.1101/2020.06.03.132241.
109. Keating, S.E.; Griffing, A.H.; Nielsen, S.V.; Scantlebury, D.P.; Gamble, Tony. Conserved ZZ/ZW sex chromosomes in Caribbean croaking geckos (*Aristelliger*: Sphaerodactylidae). *J. Evol. Biol.* **2020**, *33*, 1316-1326. doi: 10.1111/jeb.13682.
110. Gornung, E.; Mosconi, F.; Annesi, F.; Castiglia, R. The first cytogenetic description of *Euleptes europaea* (Gené, 1839) from Northern Sardinia reveals the highest diploid chromosome number among sphaerodactylid geckos (Sphaerodactylidae, Squamata). *Comp. Cytogenet*. **2013**, *7*, 153. https://doi.org/10.3897/compcytogen.v7i2.4881
111. McBee, K.; Bickham, J.W.; Dixon, J.R. Male heterogamety and chromosomal variation in Caribbean geckos. *J. Herpetol*. **1987,** *21*, 68–71.
112. McBee, K.; Sites, J.W. Jr.; Engstrom, M.D.; Rivero-Blanco, C.; Bickham, J.W. Karyotypes of four species neotropical gekkos. *J. Herpetol.* **1984,** *18*, 83–84.
113. Gamble, T.; McKenna, E.; Meyer, W.; Nielsen, S.V.; Pinto, B.J.; Scantlebury, D.P.; Higham, T.E. XX/XY sex chromosomes in the South American dwarf Gecko (*Gonatodes humeralis*). *J. Heredity* **2018**, *109*, 462-468; https://doi.org/10.1093/jhered/esx112.
114. Kostmann, A.; Kratochvíl, L.; Rovatsos, M. First report of sex chromosomes in plated lizards (Squamata: Gerrhosauridae). *Sex Dev*. **2021**, *1*, 1-6. doi: 10.1159/000513764.
115. Gorman, G. C. New chromosome data from 12 species of lacertid lizards. *J. Herpetol.*, **1969**, *3*, 49-54.
116. Rovatsos, M.; Vukić, J.; Altmanová, M.; Johnson Pokorná, M.; Moravec, J.; Kratochvíl, L. Conservation of sex chromosomes in lacertid lizards. *Mol. Ecol*. **2016**; *25*, 3120-3126. doi: 10.1111/mec.13635.
117. Rovatsos, M.; Vukić, J. Mrugała, A.; Suwala, G.; Lymberakis, P.; Kratochvíl, L. Little evidence for switches to environmental sex determination and turnover of sex chromosomes in lacertid lizards. *Sci. Rep*. **2019**, *9*, 7832. https://doi.org/10.1038/s41598-019-44192-5.
118. Olmo, E.; Odierna, G.; Capriglione, T. Evolution of sex-chromosomes in lacertid lizards. *Chromosoma*, **1987**, *96*, 33-38. https://doi.org/10.1007/BF00285880.

119. Giovannotti, M.; Nisi Cerioni, P.; Slimani, T.; Splendiani, A.; Paoletti, A.; Fawzi, A.; Olmo, E.; Caputo Barucchi, V. Cytogenetic characterization of a population of *Acanthodactylus lineomaculatus* Duméril and Bibron, 1839 (Reptilia, Lacertidae), from Southwestern Morocco and insights into sex chromosome evolution. *Cytogenet. Genome Res.* **2017**, *153*, 86-95. doi: 10.1159/000484533.
120. Suwala, G.; Altmanová, M.; Mazzoleni, S.; Karameta, E.; Pafilis, P.; Kratochvíl, L.; Rovatsos, M. Evolutionary Variability of W-Linked Repetitive Content in Lacertid Lizards. *Genes* **2020**, *11*, 531. doi:10.3390/genes11050531.
121. Odierna, G.; Kupriyanova, L.A.; Capriglione, T.; Olmo, E. Further data on sex chromosomes of Lacertidae and a hypothesis on their evolutionary trend. *Amphib.-Reptil.* **1993**, *14,*1-11.
122. Volobouev, V.; Pasteur, G.; Bons, J.; Guillaume, C.P.; Dutrillaux, B. Sex chromosome evolution in reptiles: divergence between two lizards long regarded as sister species, *Lacerta vivipara* and *Lacerta andreanskyi*. *Genetica*, **1990**, *83,* 85–91. DOI: 10.1007/BF00774693
123. Darevsky, I.S.; Kupriyanova, L.A.; Bakradze, M.A. Occasional males and intersexes in parthenogenetic species of caucasian rock lizards (genus *Lacerta*). *Copeia*, **1978**, *2,* 201-207.
124. Kupriyanova, L.A. Diversity in parthenogenetic lacertid lizards: cytogenetic studies. In Proceedings of the 6th Ordinary General Meeting of the Societas Europaea Herpetologica; Korsós, Z., Kiss, Z., Eds.; Societas Europaea Herpetologica, Budapest, Hungary, **1992,** pp. 273-279.
125. Darevsky, I.S.; Kulikova, V.N. Naturliche Parthenogenese in der polymorphen Gruppe der Kaukasischen Felseidechse (*Lacerta saxicola* Eversmann). *Zool. Jb. Abt. Syst*., **1961**, *89,* 119-176.
126. Kupriyanova, L.A. Karyological analysis of lizards of subgenus *Archaeolacerta*. *Tsitologiya,* **1969***, 2*, 803-814.
127. Spangenberg, V.; Arakelyan, M.; Galoyan, E.; Matveevsky, S.; Petrosyan, R.; Bogdanov, Y.; Danielyan, F.; Kolomiets, O. Reticulate Evolution of the Rock Lizards: Meiotic Chromosome Dynamics and Spermatogenesis in Diploid and Triploid Males of the Genus *Darevskia. Genes* **2017**, *8*, 149; doi:10.3390/genes8060149.
128. Kupriyanova L.A. Cytogenetic evidence of genome interaction in hybrid *Lacerta*. In *Evolution and Ecology of Unisexual Vertebrates*, Dawley, R., Bogart, J., Eds.; Bull., N.Y. Museum, Albany, New York, US, 1989**,** Vol. 466, pp. 236-239.
129. Kupriyanova, L.A. Diversity in parthenogenetic lacertid lizards: cytogenetic studies. **1992**. In: Proceedings of the Sixth Ordinary General meeting Societas Europaea Herpetologica, Budapest, Hungary, 1991, Korsos, Z., Kiss, I., Eds.; SEH, Budapest, Hungary, pp. 273-279.
130. Capula, M.; Lapini, L. The karyotype of Lacerta mosorensis (Reptilia: Lacertidae): Evidence for a new case of female heterogamety in a lacertid lizard. *Rend. Fis. Acc. Lincei* **1991,** *s. 9, v. 2*: 51–57.
131. Ivanov, V.G.; Fedorova, T.A. Heterochromosomes in the karyotype of *Eremias arguta* Pall. *Tsitologiya*, **1973**, *15*, 762–765.
132. Kupriyanova, L.A. Structure, localization and stability of chromosomes in karyotype evolution in lizards of the Lacertidae family. *Russ. J. Herpetol*. **1994**, *1(2)*, 161–168.
133. Wang, C.; Tang, X.; Xin, Y.; Yue, F.; Yan, X.; Liu, B.; An, B.; Wang, X.; Chen, Q. Identification of sex chromosomes by means of comparative genomic hybridization in a lizard, *Eremias multiocellata*. *Zoolog Sci.* **2015**, *32*, 151–156.
134. Ivanov V.G.; Bogdanov, O.P.; Anisimova, E.; Fedorova, T.A. Studies of the karyotypes of three lizard species (Sauria, Scincidae, Lacertidae). *Tsitologiya* **1973**, 15:1291 1296.
135. Lisachov, A.P.; Galkina, S.A.; Saifitdinova, A.F.; Romanenko, S.A.; Andreyushkova, D.A.; Trifonov, V.A.; Borodin, P.M. Identification of sex chromosomes in *Eremias velox* (Lacertidae, Reptilia) using lampbrush chromosome analysis. *Comp. Cytogen*. **2019**, *13*, 121-132; doi: 10.3897/CompCytogen.v13i2.34116.
136. Olmo, E.; Odierna, G.; Cobror, O. Variazioni cromosomiche inter- e intraspecifiche nei lacertidi. *Atti Soc Tosc. Sci. Nat. Mem.,* **1985,** *Serie B92*, 311-318;
137. Baez, M.; Cano, J.; Lopez-Jurado, F. Estudios sobre la Herpetofauna Canaria I. El Cariotipo de *Gallotia simonyi stehlini* y de *G. atlantica* spp. en Poblaciones de la Isla de Gran Canaria1. Amphib.-Reptil. **1986**, *7*, 259-270.
138. Odierna, G.; Capriglione, T.; Olmo, E.; Cardone, A.; Rosati, C. The karyology of some South African lacertids belonging to the genera *Heliobolus*, *Meroles*, *Pedioplanis. J. Afr. Zool.* **1990**, *104,* 541-547.
139. Odierna, G.; Aprea, G.; Arribas, O.J.; Capriglione, T.; Caputo, V.; Olmo, E. The karyology of the Iberian rock lizards. *Herpetologica* **1996**, *52*, 542-550.
140. Rojo, V. Cytogenetic and molecular characterization of lacertid lizard species from the Iberian Peninsula. PhD thesis, Universidade da Coruña, La Coruña **2015**. Available at: http://hdl.handle.net/2183/16153.
141. Capula, M.; Lapini, L.; Capanna, E. The karyotype of *Lacerta horváthi* (Reptilia, Sauria, Lacertidae. *Genetica* **1982**, 79: 11-16. https://doi.org/10.1007/BF00056060.
142. Rojo, V.; Giovannotti, M.; Naveira, H.; Nisi Cerioni, P.; González-Tizón, A.M.; Caputo Barucchi, V.; Galán, P.; Olmo, E.; Martínez-Lage, A. Karyological characterization of the endemic iberian rock lizard, *Iberolacerta monticola* (Squamata, Lacertidae): insights into sex chromosome evolution. *Cytogenet. Genome Res*. **2014**, *142*,28-39; doi: 10.1159/000356049.
143. Olmo, E.; Odierna, G.; Capriglione, T.; den Bosch, H.A.J.I. 2001 The karyology of *Lacerta brandtii* (Reptilia, Lacertidae). *Folia Zool.*, **2001**, *50* (*3*): 193-196
144. Lisachov, A.P.; Giovannotti, M.; Pereira, J.C.; Andreyushkova, D.A.; Romanenko, S.A.; Ferguson-Smith, M.A.; Borodin, P.M.; Trifonov, V.A. Chromosome Painting Does Not Support a Sex Chromosome Turnover in *Lacerta agilis* Linnaeus, 1758. *Cytogenet. Genome Res*. **2020**, *160*, 134-140; doi: 10.1159/000506321.
145. Kupriyanova, L.A. Description of the karyotypes of three species of Lacertidae family. *Tsitologiya*, **1968**, *10*, 892-895.
146. Olmo E.; Odierna G.; Capriglione T. Cytotaxonomy of lacertid lizards. In: Proc. Fourth Ord. Gen. Meet. S.E.H., Nijmegen, Netherlands, 1987, van Gelder, J.J., Strijbosch, H., Bergers, P.J.M., Eds.; Fac. Sci. Nijmegen, Nijmegen, Netherlands, 1987, 303-306.
147. Ivanov, V.G.; Fedorova, T.A. Sex heteromorphism of chromosomes in *Lacerta strigata* Eichwald. *Tsitologiya*, **1970**, *12*, 1582–1585.
148. Gorman, G.C. New chromosome data for 12 12 species of Lacertid lizards. *J. Herpetol.,* **1969***, 3,* 49-54.
149. Olmo, E.; Odierna, G.; Capriglione, T. The karyology of Mediterranean lacertid lizards. In *Lacertids of the Mediterranean region: A biological approach*, Valakos, E.D., Bohme, W., Perez Mellado, V., Maragou, P., Eds.; Hellenic zoological society, Athens, Bonn, Alicante, 1993, pp 61-84.
150. Fritz, B.; Bischoff, W.; Fritz, J.P. Karyologische Untersuchungen an der Omaneidechse *Lacerta jayakari* BOULENGER, 1887. *Bonn. Zool. Beitr.* **1991**, *42*, 67-73.
151. Bhatnagar, A.U.; Yoniss, Y.T. A proof of female heterogamety in a lacertid lizard Ophisops elegans. *Cytologia* **1976**, *41*, 507–511.
152. Bosch, H.A.J. in den, Odierna, G., Aprea, G., Barucca, M., Canapa, A., Capriglione, T., Olmo, E. "Karyological and genetic variation in Middle Eastern lacertid lizards, *Lacerta laevis* and the *Lacerta kulzeri* complex: a case of chromosomal allopatric speciation. *Chrom. Res*. **2003**, *11*, 165-178. DOI: 10.1023/a:1022872016503
153. Cano, J. 1984 in Olmo, E.; Signorino, G.G. Chromorep: A Reptile Chromosomes Database. **2005**. Available online: http://chromorep.univpm.it (last accessed on 09 August 2021).
154. Odierna G.; Capriglione T.; Cardone A.; Olmo, E. I meccanismi cromosomici coinvolti nel diffrenziamento dei cromosomi sessuali dei lacertidi, In 53° Congr. dell'U.Z.I., Palermo, Italy, 1990, Unione Zoologica Italiana, Palermo, Italy, 1990, pp. 303-304.
155. De Smet, W.H.O. Description of orcein stained karyotypes of 36 lizard species (Lacertilia, Reptilia) belonging to the families Teiidae, Scincidae, Lacertidae, Cordylidae and Varanidae (Autarchoglossa). Acta Zool Pathol Antverp 1981, 76, 73-118
156. Matthey, R. Chromosomes de Reptiles: Sauriens, Ophidiens, Cheloniens. L'evolution de la formule chromosomiale chez les - Sauriens. *R. Suisse Zool*. **1931**, *38*, 117-186.
157. Capula, M.; Nascetti, G.; Capanna, E. Chromosome uniformity in Lacertidae: new data on four Italian species. *Amphib. -Reptil.* **1982***, 3,* 207-212.

158. Orlova, V. F.; Orlov, N.F. Chromosome complements and some questions of systematics of lizards of the genus *Lacerta*. *Zool. Zh*. **1969**, *48*, 1056-1060.
159. Capriglione, T.; Olmo, E.; Odierna, G.; Kupriyanova, L.A. Mechanism of differentiation in the sex chromosomes of some Lacertidae. *Amphib. - Reptil.*, **1994**, *15*, 1-8.
160. Matthey, R. La loi de Robertson et la formule chromosomiale chez deux Lacertiens: *Lacerta ocellata* Daud., *Psammodromus hispanicus* Fitz. *Cytologi*, **1939**, *10,* 32-39. doi.org/10.1508/cytologia.10.32
161. Mateo, J.A.; Cano, J. On the karyotype of three lacertid species: *Acanthodactylus erythrurus* (Schinz), *Lacerta schreiberi* Bedriaga and *Lacerta perspicillata* (Dum. and Bib.). *Rev. Esp. Herp.* **1991**, *5*, 141-147.
162. Olmo, E.; Cobror, O.; Morescalchi, A.; Odierna, G. Homomorphic sex chromosomes in the lacertid lizard Takydromus sexlineatus. *Heredity* **1984**, *53*, 457–459. doi.org/10.1038/hdy.1984.103.
163. Cano J.; Lopez, L.F.; Mateo J.A.; Guillame, C.P. Intraspecific variability in nucleolar organiser position in *Lacerta* (*Timon*) *lepida. Amphib.-Reptil.* **1999***, 20,* 73-77.
164. Odierna G., Heulin B., Guillaume C.P., Vogrin, N., Aprea, G, Capriglione, T., Surget-Groba Y., Kupriyanova L. A. Further analysis of the karyological variations existing within and between oviparous and viviparous forms of *Lacerta* (*Zootoca*) *vivipara*: evolutionary and biogeographic implications. *Ecography* **2001***, 24*: 332-340. doi.org/10.1159/000356049
165. Chevalier, M. Données nouvelles sur le caryotype du Lézard vivipare (Reptile, Lacertilien). Existe-t-il une hétérogamétie femelle de type Z1Z2W? *C. R. hebd. Séanc. Acad. Sci., Paris* **1969**, *268*: 2098-2100.
166. Kupriyanova, L.; Böhme, W. New data on the distribution of different forms of *Zootoca vivipara* in eastern and western Europe: chromosomal analysis. *Herpetologia Bonnensis*, **1997**, *13*, 199-205.
167. Eremchenko, M.; Panfilov, A.M.; Tsarinenko, E.I. Some aspects of cytogenetical and systematical research of some Asian species of Scincidae and Lacertidae. *Bishkek: Ilim* **1992**, p.1–181 (in Russian).
168. Dissanayake, D.S.B.; Holleley, C.E.; Hill, L.K.; O'Meally, D.; Deakin, J.E.; Georges, A. Identification of Y chromosome markers in the eastern three-lined skink (*Bassiana duperreyi*) using in silico whole genome subtraction. *BMC Genomics* **2020**, *21*, 667. https://doi.org/10.1186/s12864-020-07071-2.
169. Hill, P.; Shams, F.; Burridge, C.P.; Wapstra, E.; Ezaz, T. Differences in homomorphic sex chromosomes are associated with population divergence in sex determination in *Carinascincus ocellatus* (Scincidae: Lygosominae). *Cells* **2021**, *10*, 291. https://doi.org/10.3390/cells10020291.
170. Caputo, V.; Odierna, G. Karyological investigation on three Canarian scincid lizards genus *Chalcides* Laurenti, 1768 (Reptilia, Squamata). *J. Afr. Zool*., **1991**, *105*, 249–255.
171. Kostmann, A.; Kratochvíl, L.; Rovatsos, M. Poorly differentiated XX/XY sex chromosomes are widely shared across skink radiation. *Proc. R. Soc. B* **2021**, *288*, 20202139 https://doi.org/10.1101/2020.08.29.273524.
172. Donnellan, S.C. Chromosomes of Australian lygosomine skinks (Lacertilia: Scincidae) I. The *Egernia* group: C-banding, silver staining, Hoechst 33258 condensation analysis. *Genetica* **1991**, *83,* 207–222.
173. Ota, H.; Hikida, T.; Matsui, M.; Hasegawa, M.; Labang, D.J. NabhitabhataChromosomal variation in the scincid genus *Mabuya* and its arboreal relatives (Reptilia: Squamata). *Genetica* **1996**, 98, 87–94 (1996). https://doi.org/10.1007/BF00120222
174. Cornejo-Páramo, P.; Dissanayake, D.S.B.; Noriega, A.L.: Martínez-Pacheco, M.L.; Acosta, A.; Ramírez-Suástegui, C.; Méndez de la Cruz, F.R.; Székely, T.; Urrutia, A., Georges, A.; Cortez, D. Viviparous reptile regarded to have temperature- dependent sex determination has old XY chromosomes. *Genome Biol. Evol.* **2020**, *12*, 924-930; doi: 10.1093/gbe/evaa104.
175. Becak, M.L.; Becak, W.; Denaro, L. Chromosome polymorphism, geographical variation and karyotypes in Sauria. *Caryologia* **1972**, *25*, 313–326.
176. Hardy, G.S. The karyotypes of two scincid lizards, and their bearing on relationships in genus *Leiolopisma* and its relatives (Scincidae: lygosominae). *New Z. J. Zool*. **1979**, *6*, 609–612.
177. Hutchinson, M.N.; Donnellan, S.C. Taxonomy and genetic variation in the Australian lizards of the genus *Pseudemoia* (Scincidae: Lygosominae). *J. Nat. Hist.* 1**992**, *26*, 215–264.
178. Castiglia, R.; Bezerra, A.M.R.; Flores-Villela. O.; Annesi, F.; Muñoz, A.; Gornung, E. Comparative cytogenetics of two species of ground skinks: *Scincella assata* and *S. cherriei* (Squamata: Scincidae: Lygosominae) from Chiapas, Mexico. *Acta Herp*. **2013**, *8*, 69-73: DOI: 10.13128/Acta_Herpetol-11315.
179. Wright, J.W. Evolution of the X1X2Y sex chromosome mechanism in the scincid lizard *Scincella laterale* (Say). *Chromosoma*, **1973**, *43*, 101–108.
180. Patawang, I.; Chuaynkern, Y.; Supanuam, P.; Maneechot, N.; Pinthong, K.; Tanomtong, A. Cytogenetics of the skinks (Reptilia, Scincidae) from Thailand; IV: newly investigated karyotypic features of *Lygosoma quadrupes* and *Scincella melanosticta*, *Caryologia* **2018**, *71*, 29-34; DOI: 10.1080/00087114.2017.1402249-
181.King, M. Karyotypic studies of some Australian Scincidae (Reptilia). *Aust. J. Zool.* **1973**, *21,* 21–32.
182. Nielsen, S.V.; Pinto, B.J.; Guzmán-Méndez, I.A.; Gamble, T. First report of sex chromosomes in night lizards (Scincoidea: Xantusiidae). *J. Heredity* **2020**, *111*, 307-313; doi:10.1093/jhered/esaa007.
183. Yonenaga-Yassuda, Y.; Trefaut Rodriguesm, M.; Pellegrino, K.C.M. Chromosomal banding patterns in the eyelid-less microteiid lizard radiation: The X1X1X2X2:X1X2Y sex chromosome system in *Calyptommatus* and the karyotypes of *Psilophthalmus* and *Tretioscincus* (Squamata, Gymnophthalmidae)*. Genet. Mol. Biol*. **2005**, *28*,700-709.
184. Cole, C.J.; Dessauer, H.C.; Townsend, C.R.; Arnold, M.G. Unisexual lizards of the genus *Gymnophthalmus* (Reptilia: Teiidae) in the Neotropics: Genetics, origin and systematics. *Amer. Mus. Novitates* **1990,** *2994*, 1–29.
185. Yonenaga-Yassuda, Y.; Rodrigues, M.T. Supernumerary chromosome variation, heteromorphic sex chromosomes and banding patterns in microteiid lizards of the genus *Micrablepharus* (Squamata, Gymnophthalmidae). *Chrom Res*, **1999**, *6*, 21-29.
186. Pellegrino, K.C.M.; Rodrigues, M.T.; Yonenaga-Yassuda, Y. Chromosomal polymorphisms due to supernumerary chromosomes and pericentric inversions in the eyelid-less microteiid lizard *Nothobachia ablephara* (Squamata, Gymnophthalmidae). *Chrom Res*, **1999**, *7*, 247-254.
187. Cole, C.J.; Lowe, C.H.; Wright, J.W. Sex chromosomes in teiid whiptail lizards (genus Cnemidophorus). *Amer. Mus. Novitates* **1969**, *2395*, 1-14.
188. Rovatsos, M.; Rehák, I.; Velenský, P.; Kratochvíl, L. Shared ancient sex chromosomes in varanids, beaded lizards, and alligator lizards. *Mol. Biol. Evol*. **2019**, *36*, 1113-1120, https://doi.org/10.1093/molbev/msz024.
189. Augstenová, B.; Pensabene, E.; Kratochvíl, L.; Rovatsos, M. Cytogenetic Evidence for Sex Chromosomes and Karyotype Evolution in Anguimorphan Lizards. *Cells* **2021**, *10,* 1612. https://doi.org/10.3390/cells10071612.
190. Johnson Pokorná, M.; Rovatsos, M.; Kratochvíl, L. Sex chromosomes and karyotype of the (nearly) mythical creature, the Gila monster, *Heloderma suspectum* (Squamata: Helodermatidae). *Plos ONE* **2014**, *9*, e104716. https://doi.org/10.1371/journal.pone.0104716.
191. King, M. A case for simultaneous multiple chromosome rearrangements. *Genetica* **1982**, 58, 39-25.
192. Matsubara, K.; Sarre, S.D.; Georges, A.; Matsuda, Y.; Marshall Graves, J.A.; Ezaz, T. Highly differentiated ZW sex microchromosomes in the Australian *Varanus* species evolved through rapid amplification of repetitive sequences. *Plos ONE* **2014**, *9*, e95226. DOI: 10.1371/journal.pone.0095226

193. Iannucci, A.; Altmanová, M.; Ciofi, C.; Ferguson-Smith, M.; Milan, M.; Pereira, J.C.; Pether6, J.; Rehákvan, I.; Rovatsos, M.; Stanyon, R.; Velenský, P.; Ráb, P.; Kratochvíl, L.; Johnson Pokorná, M. Conserved sex chromosomes and karyotype evolution in monitor lizards (Varanidae). *Heredity* **2019**, *123*, 215-227; https://doi.org/10.1038/s41437-018-0179-6.
194. King. M.; King, D. Chromosomal evolution in the lizard genus Varanus (Reptilia). *Aust. J. Biol. Sci*. **1975**, *28*, 89-108.
195. Johnson Pokorná, M.; Altmanová, M.; Rovatsos, M.; Velenský, P.; Vodička, R.; Rehák, I.; Kratochvíl, L. First description of the karyotype and sex chromosomes in the Komodo dragon (*Varanus komodoensis*). *Cytogenet. Genome Res*. **2016**, *148*, 284-291. DOI: 10.1159/000447340.
196. Cole, C.J.; Gans, C. Chromosomes of *Bipes, Mesobaena*, and other Amphisbaenians (Reptilia), with comments on their evolution*. Amer. Mus. Novitates* **1987**, *2869*, 1-9.
